# No Evidence for Cross-Sectional or Longitudinal Associations between Cognitive Flexibility Performances and Nutritional Status in a Cohort of Inpatients with Anorexia Nervosa

**DOI:** 10.3390/nu16131982

**Published:** 2024-06-21

**Authors:** Lutzi Castaño, Melina Fatseas, Maylis Cuzacq, Lama Mattar, Nathalie Godart, Sylvie Berthoz

**Affiliations:** 1 Univ. Bordeaux, INCIA CNRS UMR 5287, 33000 Bordeaux, France; lutzi.castano@etu.u-bordeaux.fr (L.C.); melina.fatseas@u-bordeaux.fr (M.F.); maylis.cuzacq@outlook.com (M.C.); 2 Department of Addictology, Hôpital Haut-Lévêque, CHU Bordeaux, 33600 Pessac, France; 3 Department of Natural Sciences, School of Arts and Sciences, Lebanese American University, Chouran Beirut, Beyrouth 1102 2801, Lebanon; lamamattar@gmail.com; 4 Fondation Santé des Etudiants de France, 75014 Paris, France; nathalie.godart@fsef.net; 5 CESP, Univ. Paris-Sud, UVSQ, INSERM U 1178, Université Paris-Saclay, 94805 Villejuif, France; 6 UFR Simone Veil-Santé, Université Versailles Saint-Quentin-en-Yvelines, 78180 Montigny-le-Bretonneux, France; 7 Department of Psychiatry, Institut Mutualiste Montsouris, 75014 Paris, France

**Keywords:** anorexia nervosa, endophenotype, nutritional status, body composition, cognitive flexibility

## Abstract

Background: According to the Cognitive–Interpersonal model of anorexia nervosa (AN), the combined influence of cognitive and socio-emotional difficulties would constitute vulnerability and maintaining factors. Poor cognitive flexibility is one of the endophenotypic candidates (i.e., a trait marker) of the disorder, but few studies have examined its association with illness symptom variations, notably weight status. The study aimed to evaluate the relationships between cognitive flexibility performances and nutritional status indices (BMI; body composition) at different times of the disorder. Methods: Cross-sectional and longitudinal associations between cognitive flexibility (TAP 2.1) and nutritional status indices, along with anxious and depressive (HAD) and eating disorder (EDE-Q) symptomatology were investigated using univariate and multivariate analyses in a cohort of AN inpatients evaluated at hospital admission (N = 167) and discharge (N = 94). Results: We found no or negligible associations between nutritional status and HAD or EDE-Q scores or cognitive flexibility performances, either cross-sectionally or longitudinally. Cognitive performances did not significantly differ between the AN subtypes. Conclusions: In agreement with the Cognitive–Interpersonal model of AN, cognitive flexibility is independent of nutritional status, as well as the AN subtype. It is also independent of the levels of anxious, depressive, or ED symptomatology. A new therapeutic approach targeting cognitive flexibility and intolerance to change could benefit severely emaciated people with AN, regardless of disease subtype and level of dysphoria.

## 1. Introduction

Core symptom criteria for anorexia nervosa (AN) are a combination of fear of weight gain, extreme weight-control behaviors, and self-worth dependent on exaggerated preoccupations with body weight and/or shape. Less than half of patients achieve full recovery, approximately 50% relapse, and 20% develop a chronic course [1]. In addition to being an enduring condition, AN is associated with severe medical morbidity, high rates of suicide, and has the highest mortality rates of any psychiatric disorder. Therefore, understanding the factors that are at play in treatment-resistance remain a major concern.

Over the past two decades, a paradigm shift has occurred in the conceptualization of the etiopathogenesis of AN, with, notably, a growing interest in the identification of endophenotypes—intermediate markers between genes and behavior. By definition, an endophenotype is a heritable trait that is quantifiable irrespective of the clinical state of an affected individual, and that is more marked among first-degree non-affected relatives than in the general population [2]. This conceptualization has been integrated into the “Cognitive–Interpersonal model” of AN [3], according to which a combination of cognitive and socio-affective endophenotypes would be involved not only in the development, but also in the maintenance of the disease, whatever the subtype of AN.

In this model, one neurocognitive characteristic that seems to meet the endophenotype definition criteria is poor cognitive flexibility or set-shifting, a high-level cognitive process that is commonly defined as the ability to alternate between several mental sets, tasks, or strategies to adjust one’s behavior to new concepts, rules, or demands. Since the model was first proposed and subsequently revised, there has been a growing number of arguments suggesting poor cognitive flexibility is a characteristic of the disease, not only in the acute stage, but also after body weight normalization [4]. A recent systematic review showed that while the majority of the studies that evaluated associations between BMI and cognitive flexibility abilities in people with AN found negligible and/or no significant associations, the scarcity of studies in this research domain makes it difficult to firmly conclude this neurocognitive marker is a trait [5]. There is, notably, a critical shortage of longitudinal studies. Moreover, though BMI is a very commonly used anthropometric index, body composition assessment is considered a better proxy of an individual’s nutritional status through the quantification of the relative proportions of fat mass (FM) and fat-free mass (FFM) to the total body mass. To the best of our knowledge, no study has investigated the relationship between body composition and cognitive flexibility in people with AN.

The current study aimed to investigate the links between nutritional status in people with AN, as indexed by body composition, in addition to BMI, and cognitive flexibility at different times of the disease. In addition, we considered a range of potentially confounding factors identified in the literature as likely impacting our variable of interest [4,5,6].

In support of the Cognitive–Interpersonal model of AN, we hypothesized that in a large sample of inpatients, (i) cross-sectional associations between nutritional status and cognitive flexibility performances at hospital admission, as well as discharge, would be negligible, if any; (ii) associations between evolution in nutritional status and evolution in cognitive flexibility performances during hospitalization would be negligible, if any; and (iii) cognitive flexibility performances would be independent of the AN subtype.

## 2. Materials and Methods

### 2.1. Study Population

The study population was part of the multicentered EVHAN prospective cohort (Eudract number: 2007-A01110-53). The EVHAN inclusion criteria were: being hospitalized for AN due to a high risk to life, either physical (i.e., BMI < 15 kg/m^2^) or psychological (i.e., suicidality), and/or recent sudden, rapid, life-threatening weight loss; consent to take part in the study; and affiliation with the French Social Security system. Exclusion criteria were insufficient proficiency in French language and the presence of psychosis, and/or a potentially confounding somatic pathology (e.g., diabetes, Crohn’s disease, or other metabolic disorders). Three additional criteria were added for the present analyses: being at least 13 years of age, as younger participants underwent different assessments; only female participants, due to the very low proportion of males (4.50%); participants with no missing data on nutritional status and cognitive flexibility performances. Based on these criteria, the final sample included 167 inpatients.

### 2.2. Measures

#### 2.2.1. Self-Report Questionnaires

Levels of eating disorder symptoms were evaluated using the Eating Disorder Examination Questionnaire (EDE-Q 5.2; French version: [7]), a 28-item self-report questionnaire that assesses the frequency/severity of eating disorder symptoms over the past 28 days. Twenty-two of the items are rated on a Likert scale from 0 (no days/not at all) to 6 (everyday/markedly) and form four subscales: food restriction (5 items), preoccupation with food (5 items), preoccupation with body shape (8 items), and preoccupation with weight and the desire to lose weight (5 items). The EDE-Q total score (average of the scores for the four dimensions) is presented for descriptive purposes. It is the most commonly used self-report of ED symptomatology. In the present sample, the Cronbach alpha was 0.914 at admission and 0.942 at discharge.

Depressive and anxious symptoms were assessed using the Hospital Anxiety and Depression scale (HAD; French version [8]). It determines the presence and severity of the most common symptoms of depression (7 items) and anxiety (7 items) over the past seven days in a hospital setting. Items are rated on a 4-point Likert scale (0–3); depression and anxiety score each range between 0 (no symptoms) and 21 (severe levels of symptoms). In the current sample, Cronbach’s alpha for the depression and anxiety subscales were, respectively, 0.794 and 0.789 at admission, and 0.759 and 0.816 at discharge.

#### 2.2.2. Neuropsychological Assessment

The Test of Attentional Performance (TAP2.1) is a computerized battery of neuropsychological tests [9]. Two subtests were chosen for the present study:

- The alertness subtest was used to adjust for potential psychomotor retardation. Participants were asked to press the answer key as quickly as possible as soon as a cross appeared in the center of the screen. The cross appeared at random intervals and no feedback was provided. The variable of interest was the median reaction time (RT).
- The flexibility subtest is a set-shifting task with low learning sensitivity. The complex non-verbal condition with 100 trials was chosen to control for the potential bias of age-related differences in verbal ability. A series of angular and rounded figures appeared simultaneously on the screen (Supplementary Figure S1) and participants were required to press the right or left response key as quickly as possible depending on whether the target stimulus appeared to the right or left of the screen’s center. The instruction was to respond alternately to angular and rounded targets, starting with the angular shape. No feedback was provided. The variable of interest was the median reaction time (RT) for correct answers.

#### 2.2.3. Nutritional Status

For BMI, body weight and height were measured using, respectively, a standard weighing scale (Omega-SECA, Hamburg, Germany) and a stadiometer (wall-mounted model 222-SECA, Hamburg, Germany).

Body composition was assessed by bio-impedancemetry using the Bioelectrical Analyzer (FORANA Helios, Frankfurt, Germany) with 50 kHz alternating current and 800 mAmp, and four skin electrodes (BIANOSTIC, Datalnput, Darmstadt, Germany) positioned on the right ankle and wrist. The participant was placed in a supine position on a bed. To ensure a satisfying conductance, the skin was cleaned with 70% alcohol. Resistance (R) and reactance (Xc) in Ohms were determined. An impedance value was then measured by impedancemetry, and body composition was calculated using the Deurenberg equation as previously validated for people with anorexia nervosa [10,11]. To be independent from stature, fat mass (FM) and fat-free mass (FFM) were adjusted for height as follows for FM and FFM, respectively: FM (kg)/height (m^2^); FFM (kg)/height (m^2^).

Data collection occurred first within the first two weeks of hospitalization (referred to as admission variables in the following text), and again in the week prior hospital discharge, except for four participants who discharged against medical advice and were tested a few days after dropping out (referred to as discharge in the following text and tables). The participants were evaluated in a quiet testing room dedicated to research investigation.

### 2.3. Statistical Analyses

A descriptive analysis was performed using means, medians, and standard deviations for continuous variables, and frequency for categorical variables. Only FM, FFM, BMI, and HAD anxiety scores were normally distributed.

For four participants, FM were missing at admission. HAD and EDE-Q scores were missing at follow-up for six participants.

Comparative univariate analyses were carried out accordingly using a Wilcoxon test or a paired-sample Student’s *t*-test and associated effects size where appropriate.

For the relationships between markers of nutritional status (BMI, FM, FFM) and cognitive test performances, non-parametric univariate (Spearman’s coefficients of correlation) and multivariate (partial coefficients of correlation or rank-based regressions) associative analyses were carried out cross-sectionally and longitudinally. For the longitudinal associations, evolution was indexed using delta scores.

The Influence of the following potentially confounding factors was investigated: age, level of education, illness duration (calculated from time of significant symptom onset in the present analyses), levels of anxious and depressive symptoms, AN subtypes, and use of pharmacological treatments that may impact performance [anxiolytics, antidepressants, benzodiazepines, antipsychotics, and hypnotics]. Spearman correlation coefficients (rho), Kruskal–Wallis or Mann–Whitney tests, and associated effect sizes were used, respectively, for continuous and categorical variables.

We used the following rule of thumb for interpreting the size of a correlation coefficient [12]: negligible <0.30, low [0.30–0.50], moderate [0.50–0.70], strong >0.70. We also computed 95% Confidence Intervals (CI) for the reported effect size.

For the comparative analyses, we used the following rules of thumb for interpreting the effect size:

◾ For paired *t*-tests, Cohen’s d coefficient |d|: negligible < 0.20; small [0.20–0.50]; medium [0.50–0.80]; large >0.80 [13].
◾ For Mann–Whitney tests, r coefficient |r|: negligible < 0.10; small [0.10–0.30]; medium [0.30–0.50]; large >0.50 [14].
◾ For Wilcoxon tests or Kruskal–Wallis, ɳ^2^ coefficient: negligible < 0.01; small [0.01–0.06]; medium [0.06–0.14]; large > 0.14 [15].

A *p*-value ≤0.05 was considered significant. To control for False Discovery Rate (FDR), a Bonferroni correction was applied a posteriori by taking the number of tests and dividing it into the alpha value. Data were analyzed using SPSS-21.0 software.

## 3. Results

### 3.1. Sample Characteristics

At admission, the sample comprised of 167 participants, aged 13 to 51 (mean age = 20.61, SD = 6.35), with a mean illness duration of 4.06 years (SD = 4.0), and included 85 of the restrictive (AN-R) and 82 of the binge–purging (AN-BP) subtypes. Participants reported their highest levels of diploma and 63 (37.70%) achieved a college degree or above, 38 (22.80%) completed 12th grade of school, 39 (23.40%) completed 9th grade of school, and 27 (16.20%) completed 5th grade of school.

At hospital discharge, the sample comprised 94 participants, including 44 AN-R and 50 AN-BP, with an average length of hospital stay of 122.28 days (SD = 103.76).

Baseline clinical characteristics, self-report questionnaires, and TAP2.1 scores of the participants lost to follow-up (Supplementary Table S1) were not significantly different from those of the remaining participants, except for BMI (respectively: mean ± SD = 14.83 ± 1.69, 14.27 ± 1.29; *p* = 0.015) and FM (respectively: mean ± SD = 2.29 ± 1.24, 1.78 ± 0.95; *p* = 0.006).

Table 1 shows the descriptive and comparative statistics of the nutritional status markers, self-report questionnaires scores, and TAP2.1 performances at baseline and follow-up. See also Supplementary Histograms.

### 3.2. Identification of Potentially Confounding Variables

As expected, performances (median RTs for correct answers) during the alertness and cognitive flexibility tasks were significantly correlated at baseline (rho = 0.421; *p* < 0.001) and follow-up (rho = 0.596; *p* < 0.001). Descriptive and comparative analyses between the AN-R and AN-PB subtypes are presented in the Supplementary Table S2. The two groups had similar alertness (*p* = 0.407; η^2^ = 0.004) and cognitive flexibility performances (*p* = 0.902; η^2^ < 0.001).

Correlative analyses showed that, at both times of evaluation, BMI was significantly correlated with FFM (baseline: rho = 0.658, *p* < 0.001; follow-up: rho = 0.742; *p* < 0.001) and FM (baseline: rho = 0.781, *p* < 0.001; follow-up: rho = 0.881; *p* < 0.001), so they were investigated in separate models in the remaining analyses (Supplementary Tables S3 and S4).

Age was significantly associated with alertness at follow-up only (baseline: rho = 0.079, *p* = 0.313; follow-up: rho = 0.247; *p* = 0.017), with cognitive flexibility performances at both time points (baseline: rho = 0.274, *p* < 0.001; follow-up: rho = 0.398; *p* < 0.001), and with two of the markers of nutritional status at follow-up only (BMI baseline: rho = 0.027, *p* = 0.731; follow-up: rho = −0.241, *p* = 0.019; FM: baseline: rho = 0.081, *p* = 0.303; follow-up: rho = −0.072, *p* = 0.488; FFM: baseline: rho = −0.144, *p* = 0.064; Follow-up: rho = −0.298; *p* = 0.004) (Supplementary Tables S3 and S4).

Illness duration was significantly associated with cognitive flexibility performances (baseline: rho = 0.225; *p* = 0.003; follow-up: rho = 0.210; *p* = 0.042) and with at least one of the markers of nutritional status (BMI baseline: rho = 0.109, *p* = 0.162; follow-up: rho = 0.223, *p* = 0.031; FFM baseline: rho = −0.318; *p* < 0.001; follow-up: rho = −0.298; *p* = 0.004; FM baseline: rho = 0.068, *p* = 0.386; follow-up: rho = −0.099, *p* = 0.340) (Supplementary Tables S2 and S3). Age and illness duration were correlated (rho = 0.532; *p* < 0.001) (Supplementary Table S2), so only age was kept in the remaining analyses.

Education level was not significantly associated with neuropsychological performances at baseline (alertness H = 2.719 *p* = 0.437; cognitive flexibility (H = 7.184; *p* = 0.066), but was significantly associated with both alertness (H = 9.414; *p* = 0.024) and cognitive flexibility (H = 13.879; *p* = 0.003) performances at follow-up. Age and education level were significantly associated (H = 50.714; *p* < 0.001), so only age was kept in the remaining analyses.

Regarding pharmacological treatments (Supplementary Table S5), comparative analyses showed that use of antidepressants (baseline: *p* = 0.01), benzodiazepines (baseline: *p* = 0.001; follow-up: *p* = 0.037), and antipsychotics (baseline: *p* = 0.001; follow-up: *p* = 0.009) were significantly associated with cognitive flexibility performances.

Based on these findings and using FDR-adjusted thresholds, alertness performance, age, and use of benzodiazepines and antipsychotics were retained in the multivariate models on the associations between cognitive flexibility performances and nutritional status for both the cross-sectional and longitudinal analyses.

### 3.3. Cross-Sectional Associations

Spearman’s coefficients of correlation between alertness, cognitive flexibility performances and markers of nutritional status, EDE-Q, and HAD scores are presented in Table 2.

At baseline, cognitive flexibility performances were significantly associated with HAD anxiety scores, but with none of the nutritional status markers, nor with the EDE-Q or HAD depression scores. Overall, correlations (absolute values) ranged between 0.005 and 0.156, with the weakest values for nutritional status and the strongest for HAD anxiety scores, yet all within the range of null or negligible associations.

When alertness performances and age were entered as covariates, the pattern of associations between cognitive flexibility performances and markers of nutritional status remained unchanged, while the association with HAD anxiety scores decreased and was not significant anymore (partial rho = 0.075; *p* = 0.347).

At follow-up, cognitive flexibility performances were significantly associated with BMI (rho = −0.241; *p* = 0.019) and FFM (rho = 0.298; *p* = 0.004), but not with FM or the EDE-Q or HAD scores. Correlations (absolute values) ranged between 0.080 and 0.298, with the weakest values for EDE-Q and HAD depression scores and the strongest for FFM, yet all within the range of null or negligible associations. When alertness performances and age were entered as covariates, the strength of the associations between cognitive flexibility and BMI (partial rho = 0.021; *p* = 0.839), FFM (partial rho = 0.009; *p* = 0.934), and FM (partial rho = 0.005; *p* = 0.963) decreased, and were null and not significant anymore.

Finally, ranked-order linear regressions (Supplementary Models summary) testing the potential impact of pharmacological treatments in addition to age and alertness performances showed that age and alertness, but not pharmacological treatment, remained significant independent predictors of cognitive flexibility performances at baseline (age: β = 0.191, *p* = 0.008; alertness: β = 0.396, *p* < 0.001) and follow-up (age β = 0.242, *p* = 0.007; alertness: β = 0.538, *p* < 0.001).

### 3.4. Longitudinal Associations

There was an overall significant improvement in all the clinical and neuropsychological variables (Table 1), with large effect sizes for markers of nutritional status (BMI: −1.42; FM: −0.92; FFM: −1.15), EDE-Q (0.75), and HAD scores (0.69 for anxiety and 0.95 for depression), and medium effect sizes for alertness (0.48) and cognitive flexibility performances (0.56) in univariate analyses. When the evolution in alertness performances was taken into account, the improvement in cognitive flexibility performances was not significant anymore and the effect size was null (*p* = 0.936; ɳ_p_^2^ = 0.001).

Regarding the association between evolution in neuropsychological and clinical scores (Table 3), evolution of cognitive flexibility performances was not significantly associated with that of any of markers of the nutritional status, nor with the EDE-Q or HAD scores. Correlations (absolute value) ranged between 0.019 and 0.135, with the weakest values for nutritional status markers and the strongest for HAD depression scores, all within the range of null or negligible associations. When evolution in alertness performances and age were entered as covariates, the pattern of results remained unchanged.

### 3.5. Post Hoc Analyses

We further investigated the effect of age on neuropsychological performances and compared the adolescents and adults (Mann–Whitney tests; Supplementary Table S6a,b). Adolescence was defined as an age below 25 years old, which corresponds to neurobiological and social adolescent growth [16]. The two groups had similar alertness performances at both times of evaluation (baseline: *p* = 0.689; mean difference ± SD = −3.22 ± 20.44; follow-up: *p* = 0.901; mean difference ± SD = −2.07 ± 10.40), but adolescents were faster at the cognitive flexibility task at both times of evaluation (baseline: *p* = 0.014, ɳ^2^ = 0.04; mean difference ± SD = −114.18 ± 45.83; follow-up: *p* = 0.08, ɳ^2^ = 0.03; mean difference ± SD = −107.82 ± 55.00).

## 4. Discussion

Poor cognitive flexibility has been suggested to be a neurocognitive endophenotype of AN. However, recent reviews that have attempted to clarify whether cognitive flexibility is influenced by weight status in people with anorexia nervosa found mixed findings [4,5,17], challenging the idea that poor cognitive flexibility is a trait, which is one of the core definition criteria for an endophenotype. In addition to pointing out the limited number of studies on this Issue and the heterogeneity of measures assessing different domains of cognitive flexibility (visual, perceptual, verbal), authors of these reviews stressed the need to conduct longitudinal studies with sample sizes large enough to limit the risk for the analyses to be underpowered, and to consider that both clinical and experimental criteria may influence the findings, such as AN subtype, comorbid mood variables, and associated pharmacological treatments on the one hand, and baseline psychomotor speed and learning effects on the other.

To the best of our knowledge, the present longitudinal study in acute AN included the second largest sample size ever [17], and is the first one to use body composition in addition to BMI as markers of nutritional status. As we hypothesized, neither the cross-sectional nor the longitudinal analyses indicated an association other than negligible, if any, between cognitive flexibility performances and the BMI, fat mass, or fat-free mass parameters. Our findings are consistent with the conclusions of the two other longitudinal studies conducted in large samples of AN inpatients [18,19]. These studies used different performance-based measures of cognitive flexibility (the TMT and WSCT in Sproch et al. [18]; the Brixton test in Leppanen et al. [19]). Our findings also align with the results of a more recent small-scale longitudinal study in severe AN that found no relationship between weight gain and cognitive flexibility improvement from hospital admission to hospital discharge (n = 22) or 6 months post-discharge (n = 18) [20]. Of note, while these studies measured cognitive flexibility performances with tests that are known to be impacted by repeated assessments, one of the strengths of our study is the use of a test that is less sensitive to practice effects. Overall, and in line with the Cognitive–Interpersonal model of AN, the findings suggest that cognitive flexibility is independent of nutritional status, and that cognitive flexibility in AN fulfills the state independence definition criterion for an endophenotype [2].

Our study included both adolescent and adult inpatients, as well as a balanced proportion of the restrictive and binge–purging AN subtypes. Similarly to our study, Sproch et al.’s [18] and Leppanen et al.’s [19] samples encompassed adolescents and adults, but the authors of these two longitudinal studies of AN inpatients did not provide information regarding potential differences of cognitive flexibility linked to the AN subtypes. As expected, the participants of the present study with the restrictive subtype had greater levels of starvation at admission than those with the binge–purging subtype of AN. However, they had similar cognitive flexibility performances at both times of evaluation, which aligns with the Cognitive–Interpersonal model of AN. Nonetheless, AN subtype was defined here according to a current diagnosis, but it is common for patients with AN to progress from a restrictive to a purgative form, or even to fluctuate between these two forms, and this could be a source of heterogeneity in the research findings. Some patients present an exclusively restrictive lifetime diagnosis, and it seems to be the subtype that has been the most consistently associated with the presence of cognitive rigidity [21]. In future studies, using a lifetime diagnosis could help to determine if people with AN who remain chronically in the restrictive subtype not only have the most marked behavioral but also cognitive flexibility difficulties.

Regarding the potential role of depressive and anxious mood on neuropsychological performances, the results of our study are consistent with the conclusions from the majority of the studies enclosed in recent meta-analyses [4,5]. Moreover, and as reported also in the meta-analyses, we found no association between cognitive flexibility performances and the level of eating disorder symptomatology. However, as flexibility difficulties are considered perpetuating factors, we would expect patients with greater difficulties to present a more severe ED symptomatology and less improvement during hospitalization. This being said, a known limitation of self-report questionnaires is sensitivity to poor insight and denial of the disorder. Denial of illness severity is one of the main symptoms of AN, and its impact on the responses to such questionnaires is well-exemplified in this cohort, with some patients endorsing very low EDE-Q scores. Therefore, to clarify this issue and draw firm conclusions, future studies should use clinician-administered scales.

In our study, the patient’s age and psychomotor speed were associated with cognitive flexibility performances, and the effect was independent from that of the pharmacological treatments. Moreover, if, at first glance, there was a small but significant improvement in cognitive flexibility performances from admission to discharge (median evolution in reaction times was 71 milliseconds), this effect did not survive when the evolution in alertness performances was taken into account. These findings are in line with the Cognitive–Interpersonal model of AN and add evidence for considering cognitive flexibility as a trait construct. They also further emphasize that baseline motor speed could bias the results when using measures of task switching to index cognitive flexibility [5] and one of the strengths of this study was to control for this bias in the analyses.

As expected, age and duration of illness were highly correlated, so only age was kept in the analyses of interest. Knowing that AN is an enduring condition, with frequent relapses, it was reasonable to suspect repeated episodes of starvation to increase neuropsychological impairments. However, the post hoc analyses comparing the adolescents and adults showed that at both time points, the two groups had similar psychomotor speed accuracy. Moreover, if the adolescents were performing slightly faster at the cognitive flexibility task at both time points, the effect sizes were negligeable and evolution in performances with weight gain were comparable in the two groups. The question of differences in cognitive flexibility between younger and older people with AN has been strongly debated in the past decade, but the present results are consistent with recent large-scale studies where cognitive flexibility was measured with neuropsychological batteries, including the most common performance-based tools in the field [22]. From another point of view, it may be that people who are more resistant to change and more rigid are more likely to progress towards chronicity, but this hypothesis needs to be verified by including an assessment of motivation to change at the start of treatment, in addition to the assessment of cognitive functioning. This assumption is, however, supported in part by Saure et al.’s meta-analysis showing that cognitive flexibility difficulties are more clearly observable in enduring AN (7 years or over), but to a lesser degree when the duration of illness is shorter [21].

The main strengths of the present study were the use of several markers of nutritional status in a large sample of people with AN, with a wide range of ages, and who were evaluated at AN symptom culmination and after acute AN symptomatology had been at least partially resolved, as well as the consideration of important potentially confounding factors. Our study also had some limitations that affect the generalizability of the findings, as only people with a severe form of the disease, and all being females, were included. Moreover, if there was an overall gain in weight between the two times of evaluation, a significant proportion of the sample did not achieve weight normalization at follow-up. This circumstance could significantly impact the study’s outcomes, warranting consideration. In addition, the reliance solely on BMI and body composition as a measure of nutritional status limits the comprehensive understanding of malnutrition. FMI and FFMI fail to consider the dynamics, history, or severity of malnutrition. Furthermore, the BIA method used in this study remains limited in anorexia nervosa (AN) and necessitates careful interpretation [10,11].

## 5. Conclusions

Overall, the findings support the Cognitive–Interpersonal model of AN in its cognitive aspect, and, more specifically, the endophenotypic criterion of independence from the nutritional status and AN subtype. From a research perspective, the effect of age and/or duration of the disorder remains to be clarified in future studies. In terms of treatment implication, cognitive remediation therapy (CRT) for anorexia nervosa is an intervention that has been designed to target poor cognitive flexibility and central coherence (the other cognitive endophenotypic candidate of AN), as well as their potential counter-parts in daily life, such as intolerance for changes in ED routines, stereotyped behaviors, erroneous beliefs, and ‘jumping to conclusions’. It was initially developed for adults and progressively adapted for younger individuals. Based on our findings, CRT could benefit severely emaciated people with AN, regardless of the disease’s subtype and the individual’s level of dysphoria.

Future research should use alternative indicators that account for the dynamics of weight loss and incorporate biochemical assessments to enhance the understanding of the links between nutritional and cognitive status in individuals with AN. Moreover, a broader sample encompassing varying degrees of AN severity would provide a more nuanced perspective.

## Figures and Tables

**Table 1:** BMI: Body Mass Index, FFM: fat-free mass, FM: fat mass, EDE-Q: Eating Disorders Examination Questionnaire total score, HAD: Hospital Anxiety and Depression scale, Alert.: alertness performances, Cog. Flex.: cognitive flexibility performances, evolution: discharge minus admission scores/values, ^1^ paired *t*-test *p* < 0.05, ^2^ Wilcoxon *p* < 0.05, ^3^ Cohen’s d, ^4^ Wilcoxon effect size (r).

	Admission (N = 167)		Discharge (N = 94)		Evolution	Paired Sample Analyses	Effect Size
	Mean (SD)/Median	Min–Max	Mean (SD)/Median	Min–Max	Mean (SD)/ Median	Z or t; *p*	Cohen d or r
BMI	14.52 (1.50)/14.30	10.72–18.59	17.15(2.03)/17.40	13.08–21.50	2.87 (2.08)/2.92	−16.11 ^1^; <0.001	−1.24 ^3^
FFM	12.57 (0.84)/12.51	10.39–15.37	13.56 (1.02)/13.51	11.56–16.17	1.02 (0.93)/1.05	−10.07 ^1^; <0.001	−0.92 ^3^
FM	2 (1.11)/1.85	0.11–5.10	3.51 (1.37)/3.75	0.17–6.99	1.77 (1.34)/1.63	−12.38 ^1^; <0.001	−1.15 ^3^
EDE-Q total	3.29 (1.32)/3.47	0–5.51	2.05 (1.33)/1.90	0–4.80	5.16 (5.26)/4.06	−6.99 ^2^; <0.001	0.75 ^4^
HAD anxiety	12.57 (4.29)/13	2–21	9.44 (4.17)/10	0–18	−3.35 (4.59)/−3.50	8.20 ^1^; <0.001	0.69 ^3^
HAD depression	9.25 (4.36)/9	0–18	5.02 (3.84)/4	0–16	−4.14 (4.05)/−4	11.22 ^1^; <0.001	0.95 ^3^
Alert. (ms)	273.10 (101.10)/253	185–1235	249.72(39.47)/243	175–377	−31.33 (111.64)/−14	−4.64 ^2^; <0.001	0.48 ^4^
Cog. Flex. (ms)	752.30 (191.90)/709	417–1517	682.61(185.92)/644	363–1242	−79.18 (128.81)/−71	−5.45 ^2^; <0.001	0.56 ^4^

**Table 2:** BMI: Body Mass Index, FFM: fat-free mass, FM: fat mass, EDE-Q: Eating Disorders Examination Questionnaire total score, HAD: Hospital Anxiety and Depression scale, Alert.: alertness performances, Cog. flex.: cognitive flexibility performances.

	Admission		Discharge	
	Alert. (ms) rho; *p* [95%CI]	Cog. Flex (ms) rho; *p* [95%CI]	Alert. (ms) rho; *p* [95%CI]	Cog. Flex (ms) rho; *p* [95%CI]
BMI	−0.150; 0.054 [−0.299–0.007]	−0.063; 0.415 [−0.218–0.094]	−0.368; <0.001 [−0.436–−0.088]	−0.241; 0.019 [−0.397–−0.037]
FFM	−0.167; 0.031 [−0.315–−0.011]	−0.005; 0.953 [−0.161 –0.152]	−0.407; <0.001 [−0.568–−0.225]	−0.298; 0.004 [−0.470–−0.089]
FM	−0.033; 0.680 [−0.190–0.126]	−0.081; 0.303 [−0.236–0.078]	−0.237; 0.021 [−0.393–0.003]	−0.141; 0.174 [−0.340–0.069]
HAD anxiety	0.111; 0.154 [−0.046–0.262]	0.156; 0.044 [0.000–0.305]	0.261; 0.014 [0.052–0.421]	0.189; 0.078 [0.006–0.387]
HAD depression	0.149; 0.055 [−0.008–0.298]	0.092; 0.237 [−0.065–0.245]	0.198; 0.064 [−0.066–0.320]	0.135; 0.212 [−0.023–0.362]
EDE-Q	−0.061; 0.437 [−0.215–0.097]	−0.025; 0.751 [−0.180–0.132]	0.031; 0.774 [−0.173–0.219]	0.080; 0.459 [−0.132–0.262]

**Table 3:** BMI: Body Mass Index, FFM: fat-free Mass, FM: fat mass, EDE-Q: Eating Disorders Examination Questionnaire total score, HAD: Hospital Anxiety and Depression scale, Alert.: alertness performances, Cog. flex.: cognitive flexibility performances. Δ: discharge minus admission scores/values.

	Evolution from Admission to Discharge	
	Δ Alert. (ms) rho; *p* [95%CI]	Δ Cog. Flex (ms) rho; *p* [95%CI]
Δ BMI	−0.118; 0.257 [−0.307–0.062]	0.033; 0.754 [−0.157–0.221]
Δ FFM	−0.183; 0.078 [−0.371–0.027]	0.098; 0.346 [−0.085–0.323]
Δ FM	−0.022; 0.833 [−0.231–0.179]	0.019; 0.855 [−0.216–0.199]
Δ HAD anxiety	0.105; 0.330 [0.020–0.395]	0.086; 0.426 [−0.161–0.235]
Δ HAD depression	−0.022; 0.842 [−0.051–0.334]	0.135; 0.209 [−0.198–0.198]
Δ EDE-Q	0.023; 0.828 [−0.248–0.144]	−0.029; 0.791 [−0.176–0.220]

## Data Availability

The data presented in this study are available upon reasonable request from the corresponding author. The data are not publicly available due to specifications from the study sponsor.

## Supplementary Materials

The following supporting information can be downloaded at: https://www.mdpi.com/article/10.3390/nu16131982/s1.
