# Supplementary material for: No Evidence for Cross-Sectional or Longitudinal Associations between Cognitive Flexibility Performances and Nutritional Status in a Cohort of Inpatients with Anorexia Nervosa
Source: Nutrients. 2024 Jun 21;16(13):1982. doi: 10.3390/nu16131982 (PMC11243233; doi:10.3390/nu16131982)
Supplement: Supplementary file 1 [file nutrients-16-01982-s001.zip › nutrients-3046752-supplementary.pdf]

SUPPLEMENTARY MATERIAL

Title: No evidence for cross-sectional or longitudinal associations between cognitive flexibility performances and nutritional status in a cohort of inpatients with anorexia nervosa.

Authorship:
Lutzi Castaño, Melina Fatseas, Maylis Cuzacq, Lama Mattar, Nathalie Godart and Sylvie Berthoz

Supplementary Figure

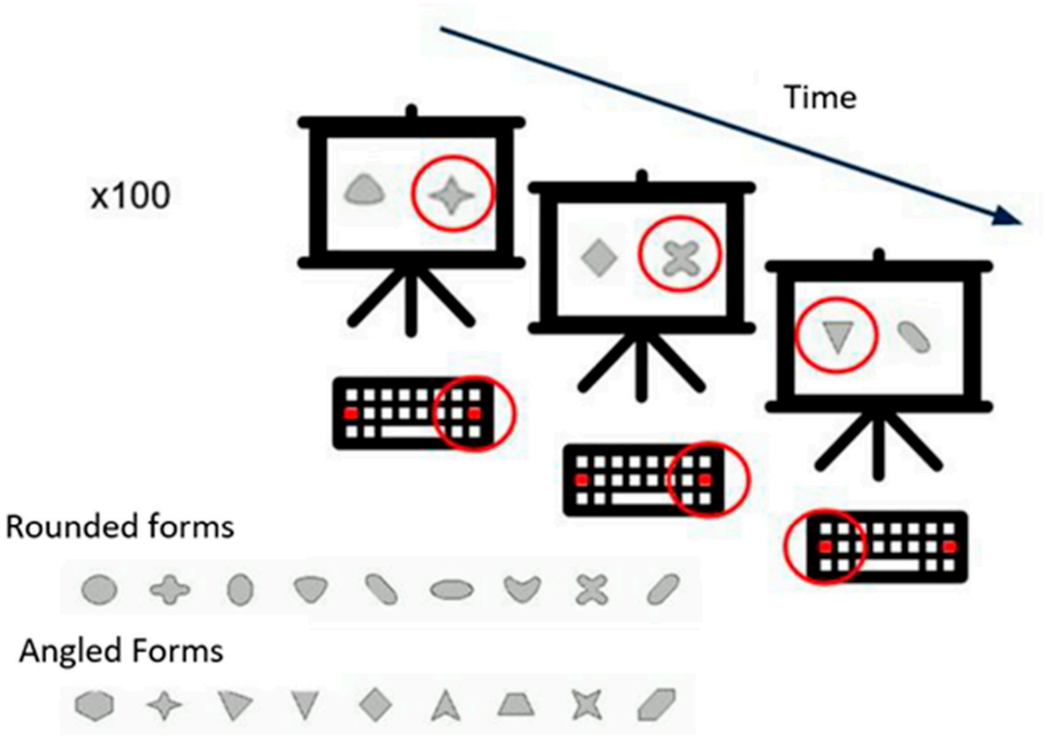

Figure S1: Illustration of the TAP 2.1 Flexibility subtest

SUPPLEMENTARY HISTOGRAMS

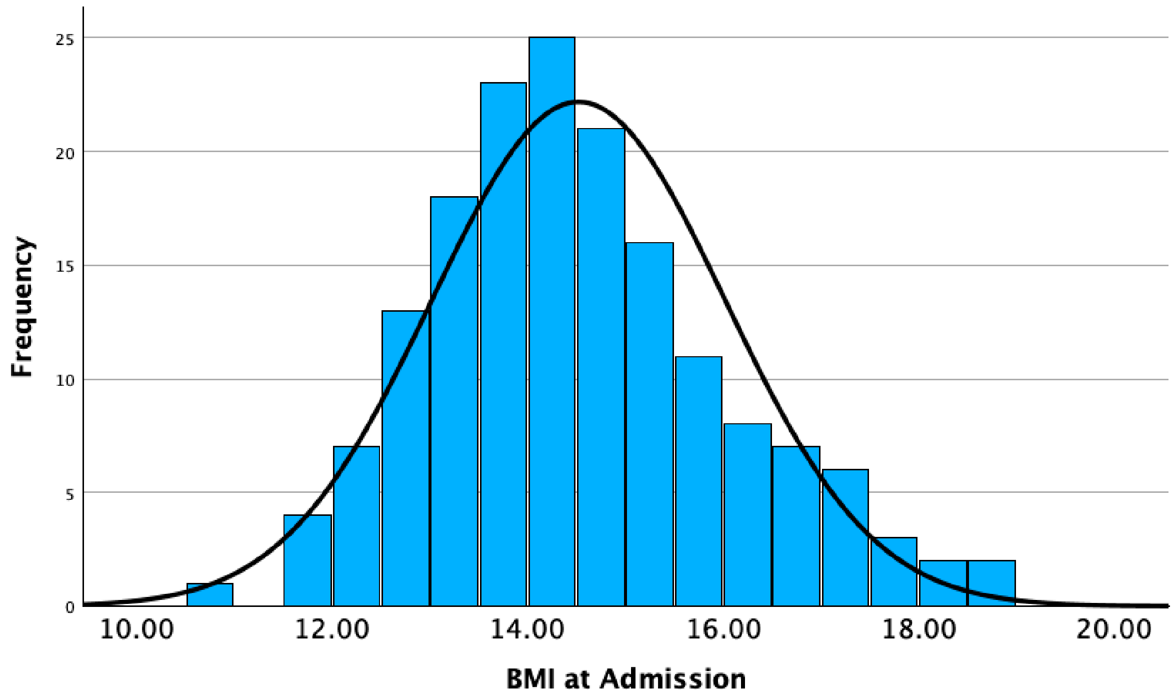

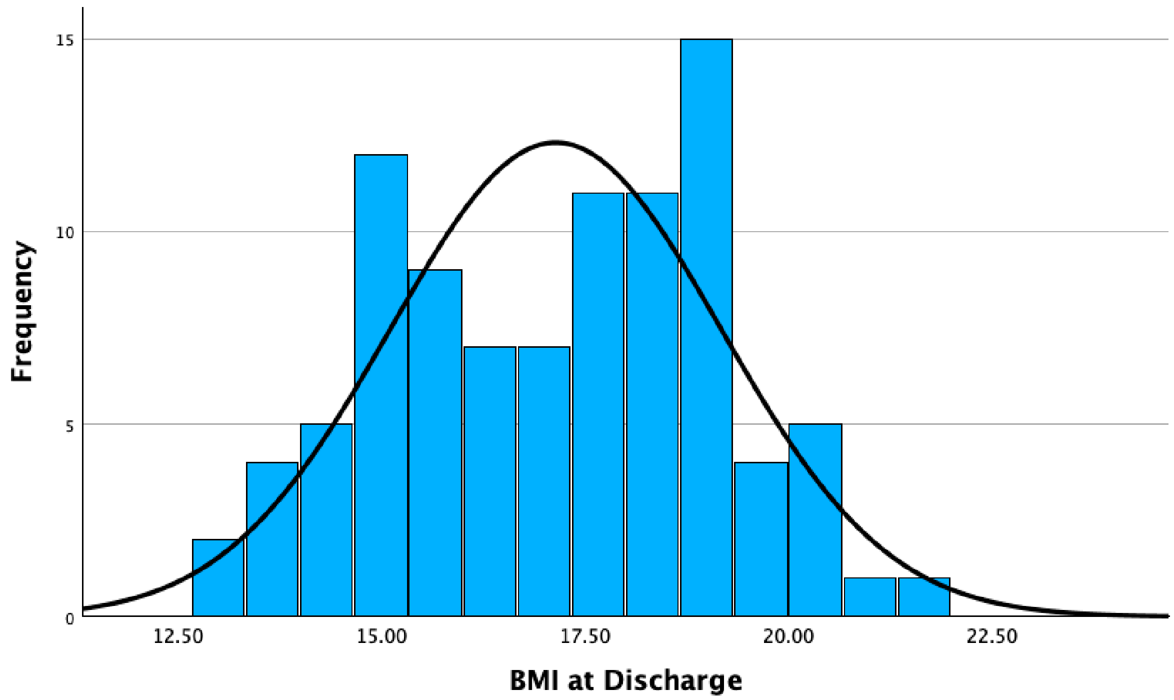

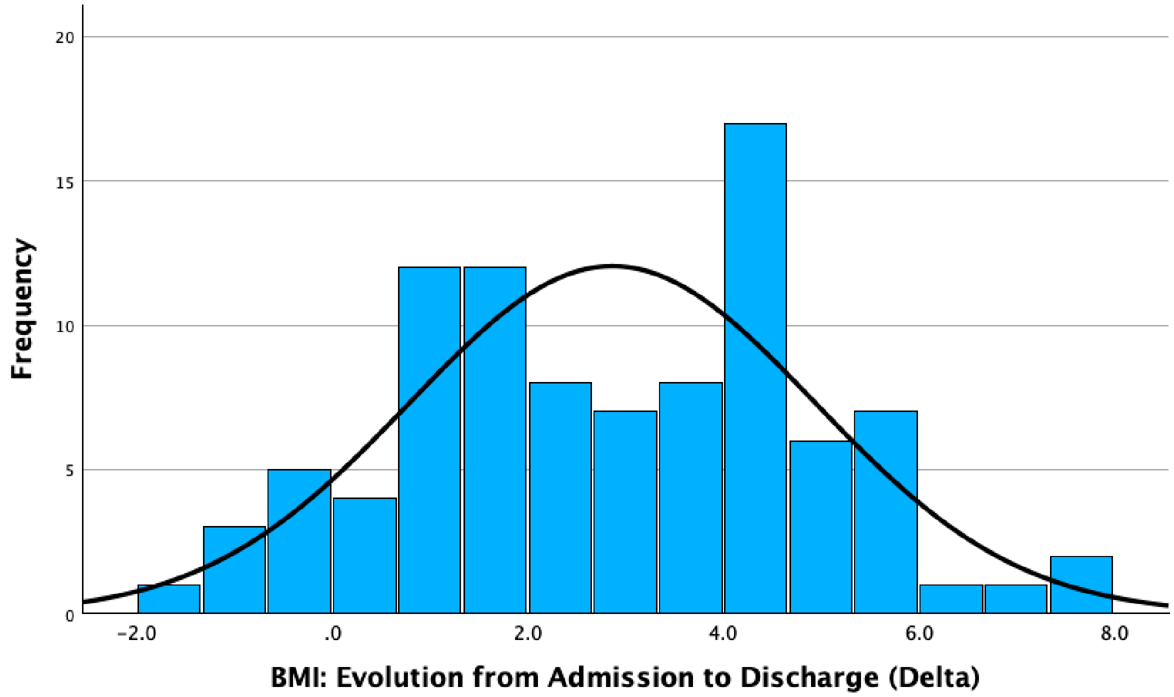

Frequency
20
15
10
5
0
-2.0
.0
2.0
4.0
6.0
8.0
BMI: Evolution from Admission to Discharge (Delta)

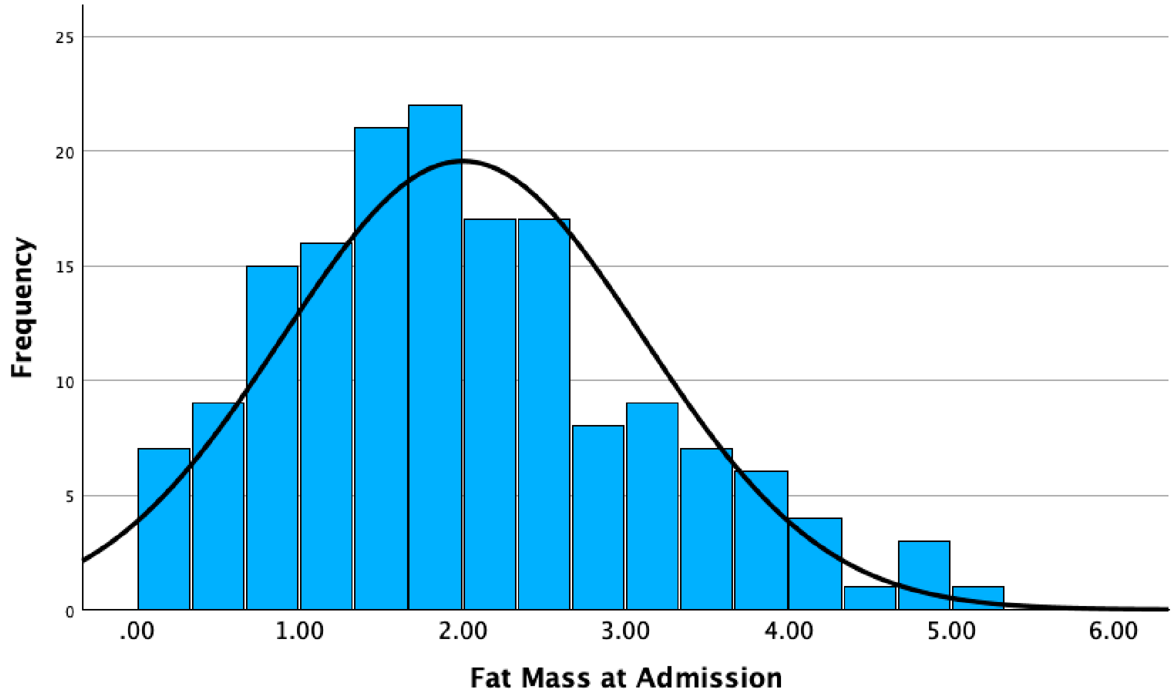

Frequency
25
20
15
10
5
0
.00
1.00
2.00
3.00
4.00
5.00
6.00
Fat Mass at Admission

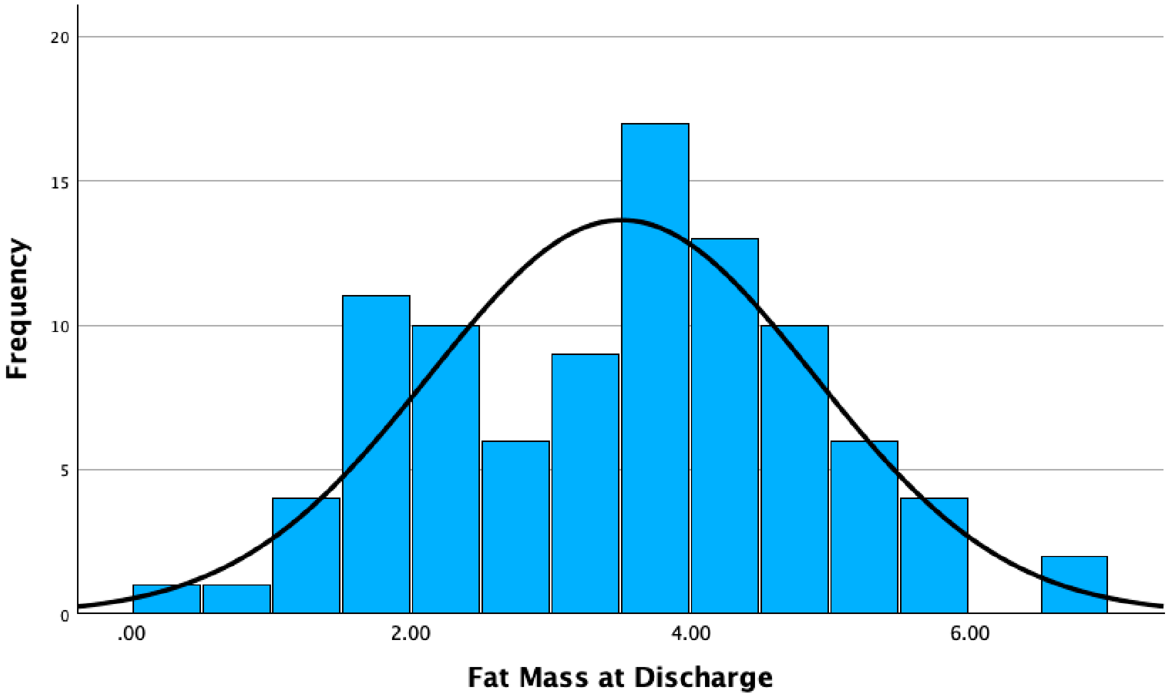

20
15
10
5
0
Frequency
.00
2.00
4.00
6.00
Fat Mass at Discharge

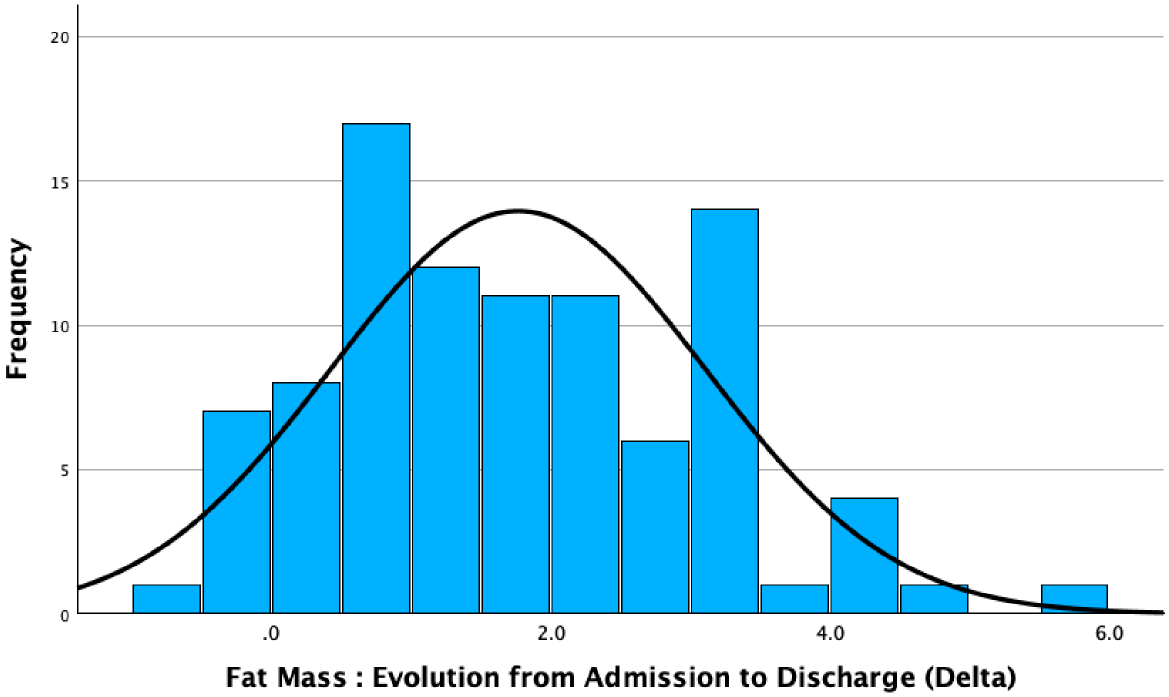

20
15
10
5
0
Frequency
.0
2.0
4.0
6.0
Fat Mass : Evolution from Admission to Discharge (Delta)

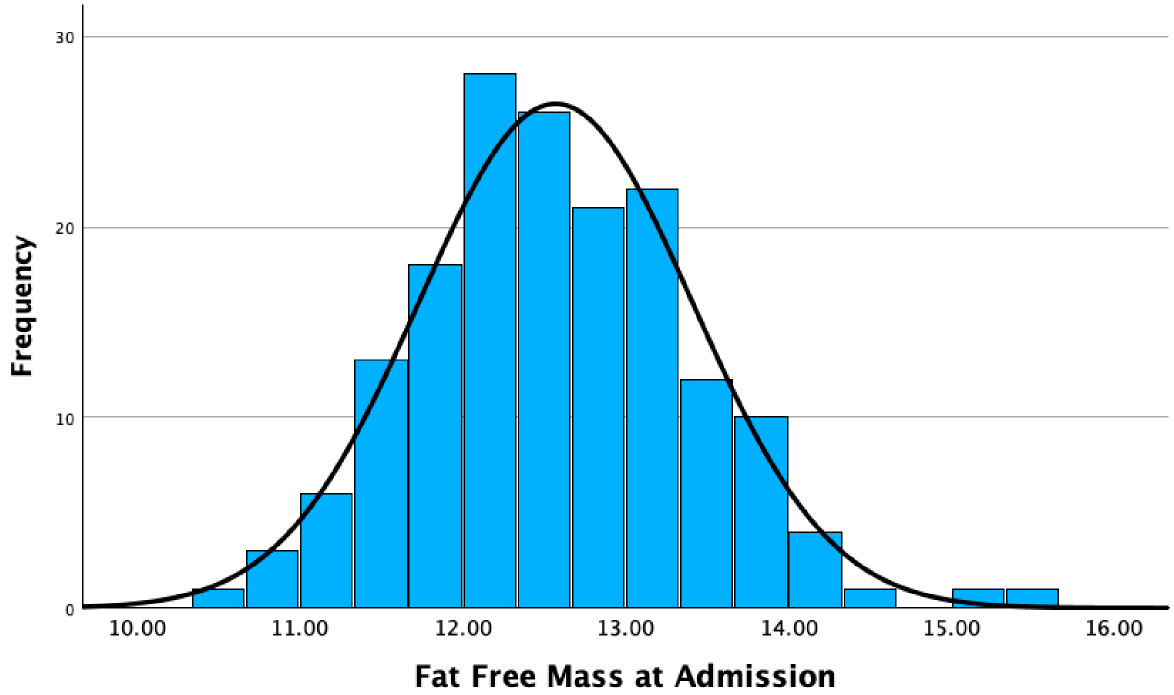
30
20
10
0
Frequency
10.00
11.00
12.00
13.00
14.00
15.00
16.00
Fat Free Mass at Admission

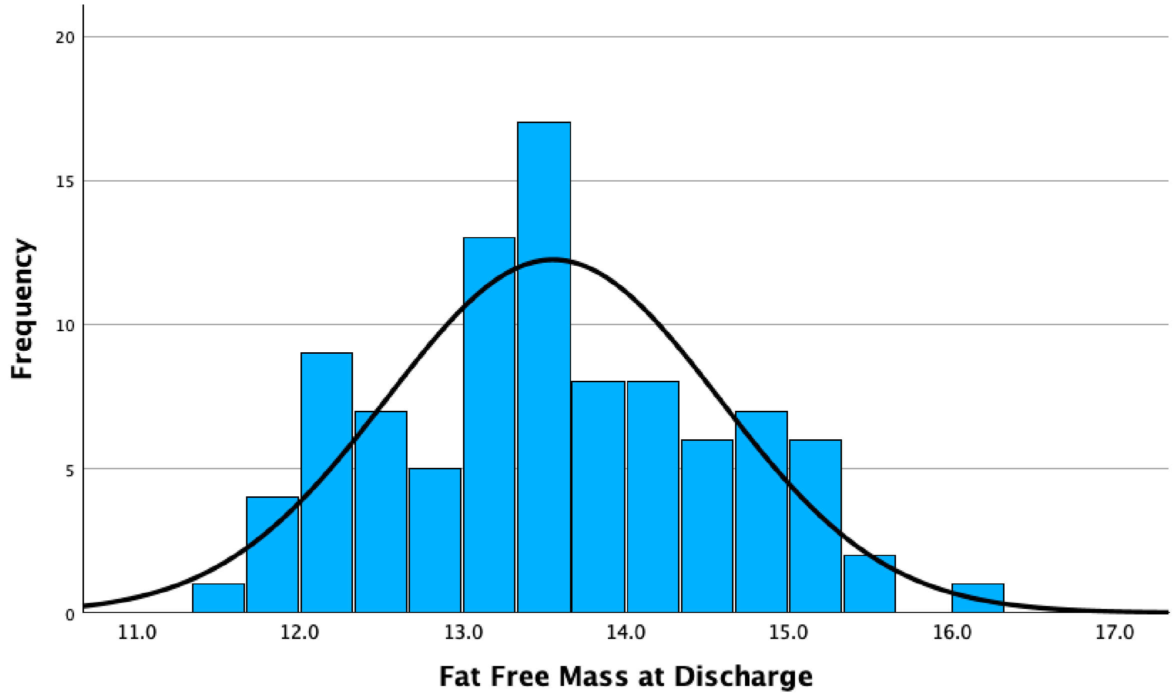
20
15
10
5
0
Frequency
11.0
12.0
13.0
14.0
15.0
16.0
17.0
Fat Free Mass at Discharge

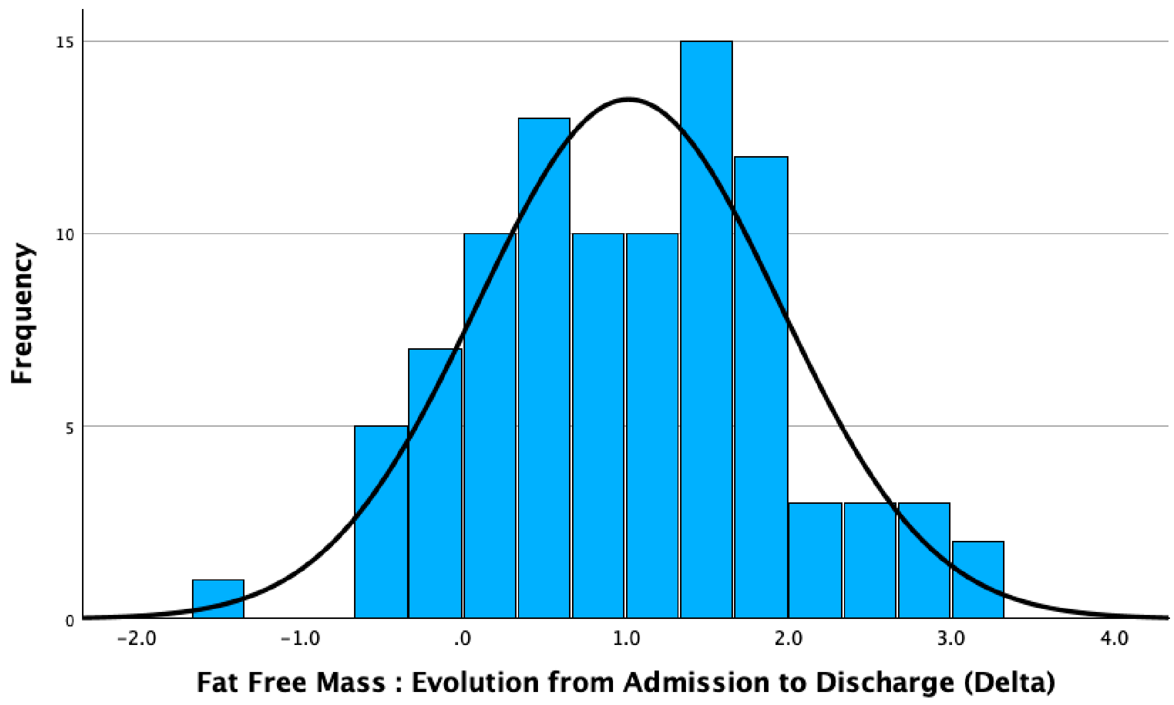
15
10
5
0
Frequency
-2.0
-1.0
.0
1.0
2.0
3.0
4.0
Fat Free Mass : Evolution from Admission to Discharge (Delta)

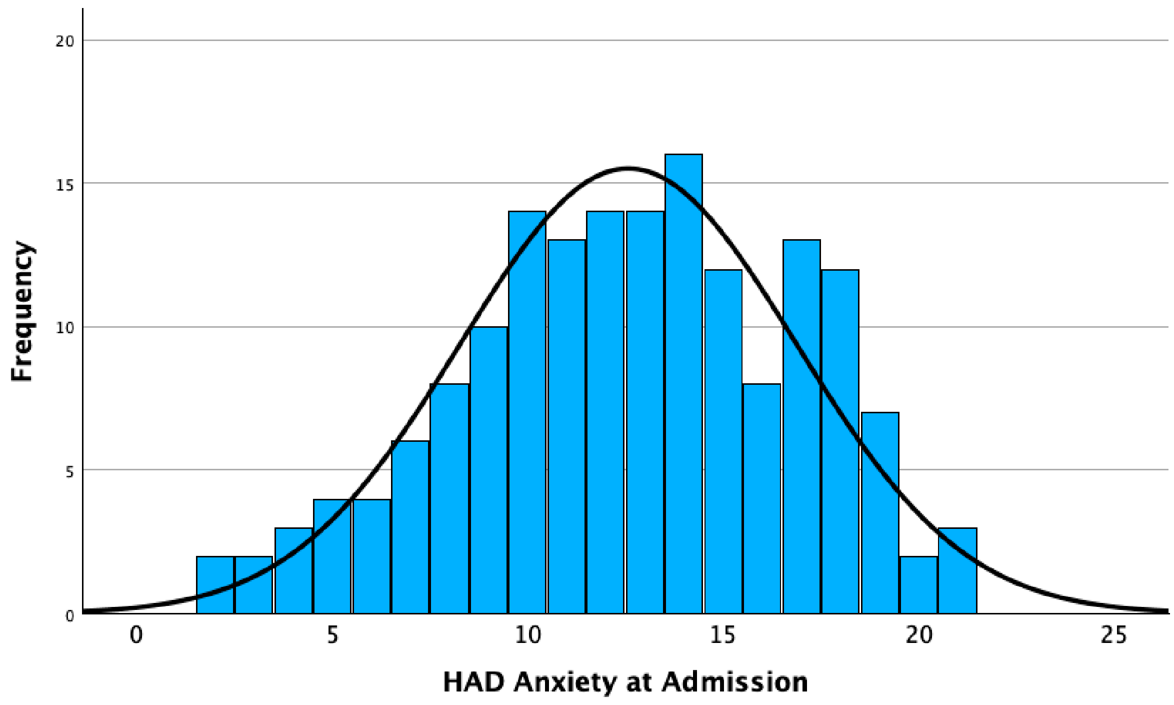
20
15
10
5
0
Frequency
0
5
10
15
20
25
HAD Anxiety at Admission

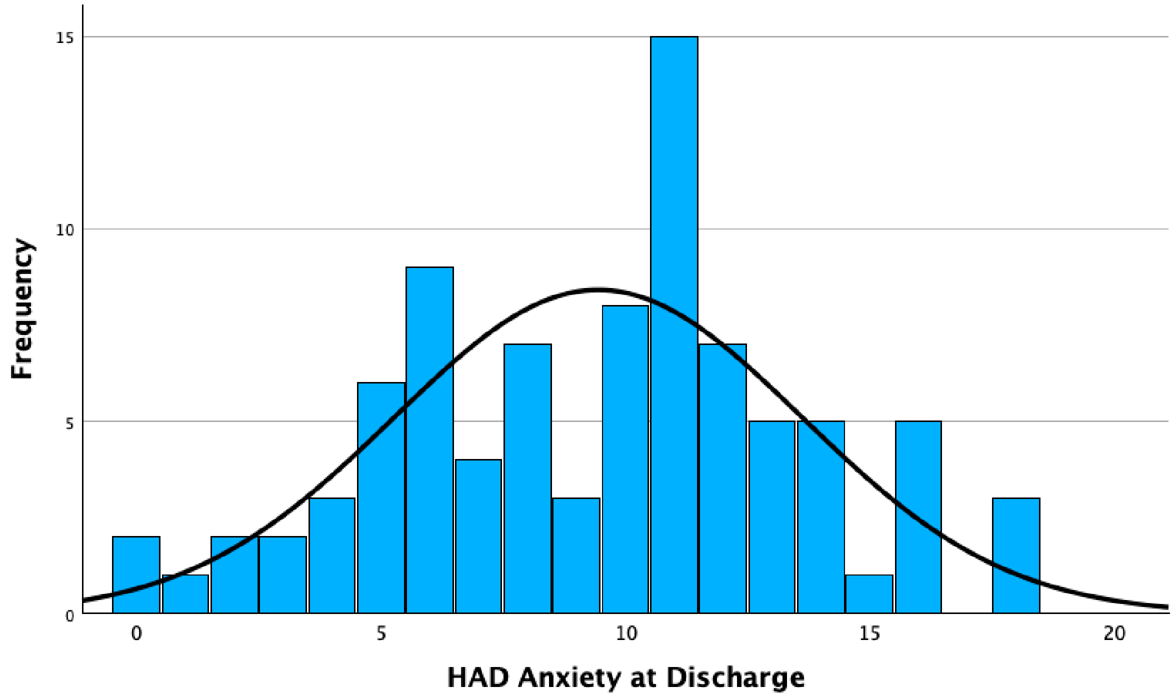
Frequency
15
10
5
0
0
5
10
15
20
HAD Anxiety at Discharge

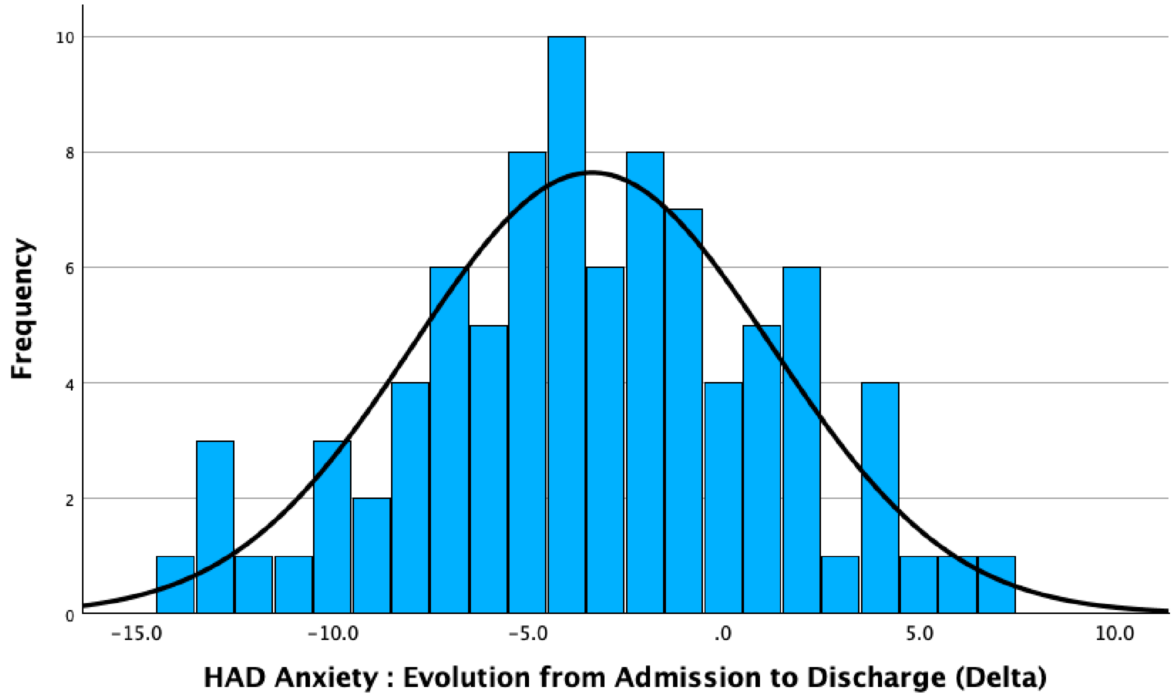
Frequency
10
8
6
4
2
0
-15.0
-10.0
-5.0
.0
5.0
10.0
HAD Anxiety : Evolution from Admission to Discharge (Delta)

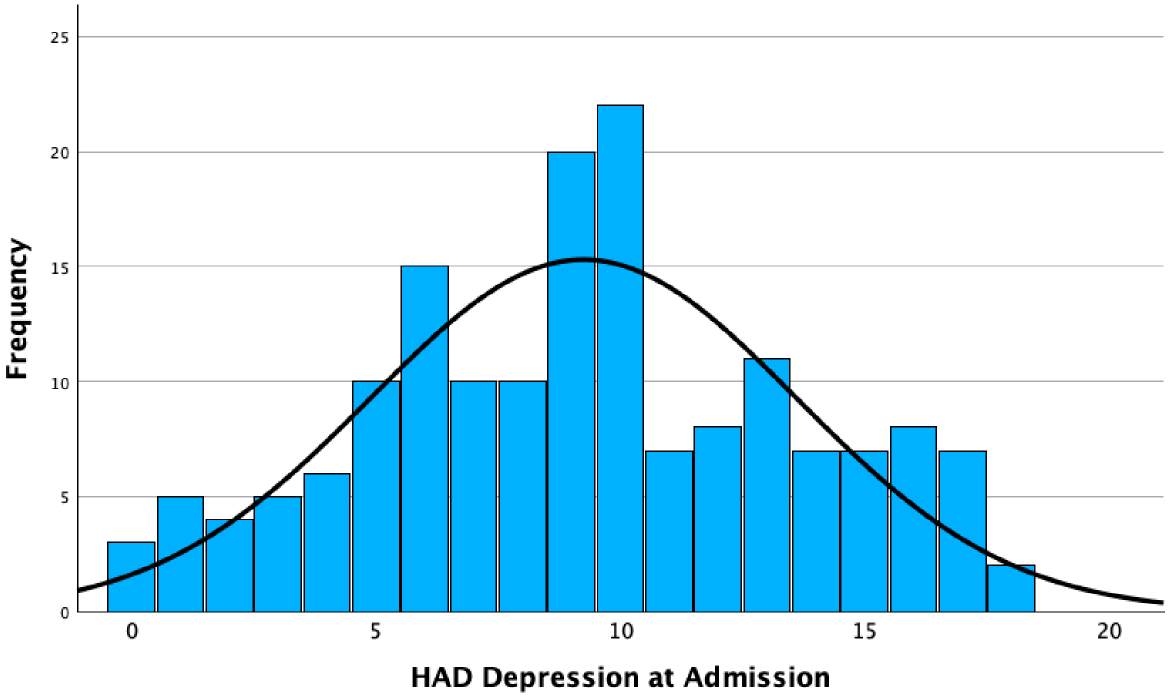

Frequency
25
20
15
10
5
0
0
5
10
15
20
HAD Depression at Admission

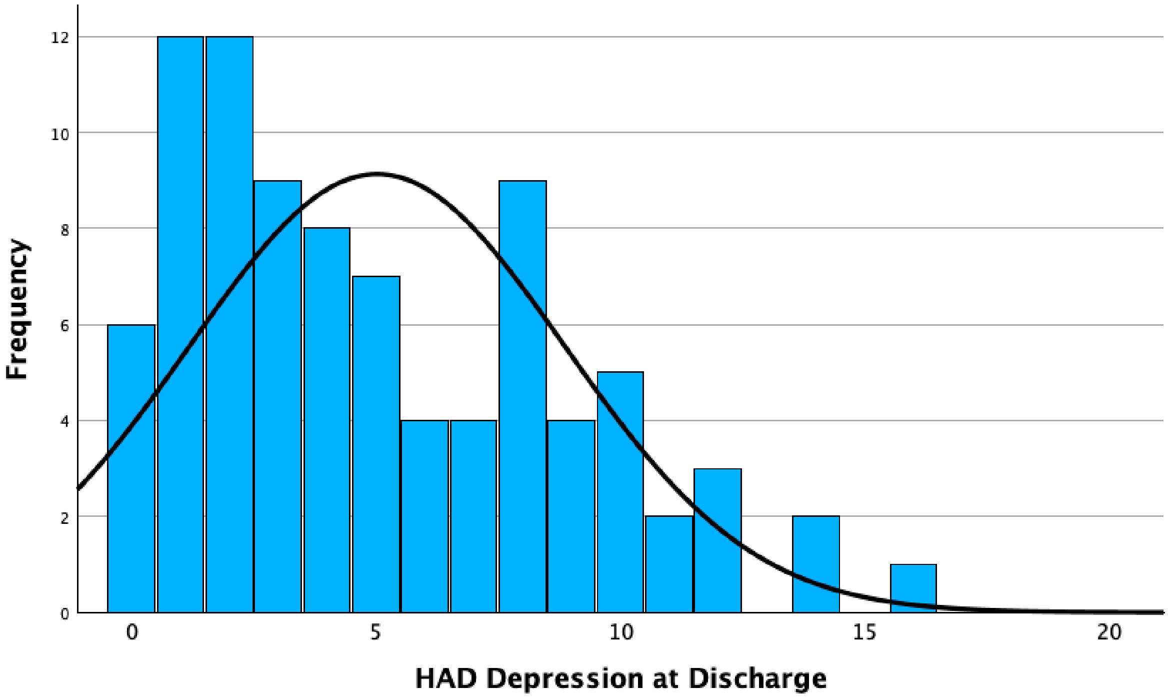

Frequency
12
10
8
6
4
2
0
0
5
10
15
20
HAD Depression at Discharge

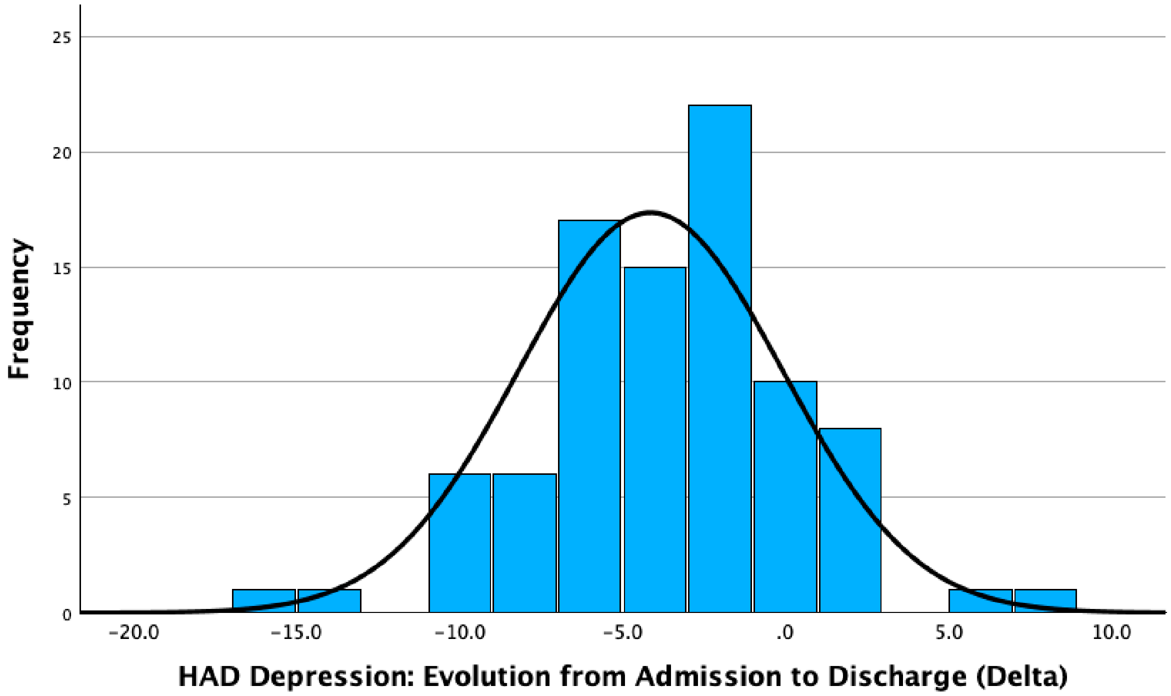

25
20
15
10
5
0
Frequency
-20.0
-15.0
-10.0
-5.0
.0
5.0
10.0
HAD Depression: Evolution from Admission to Discharge (Delta)

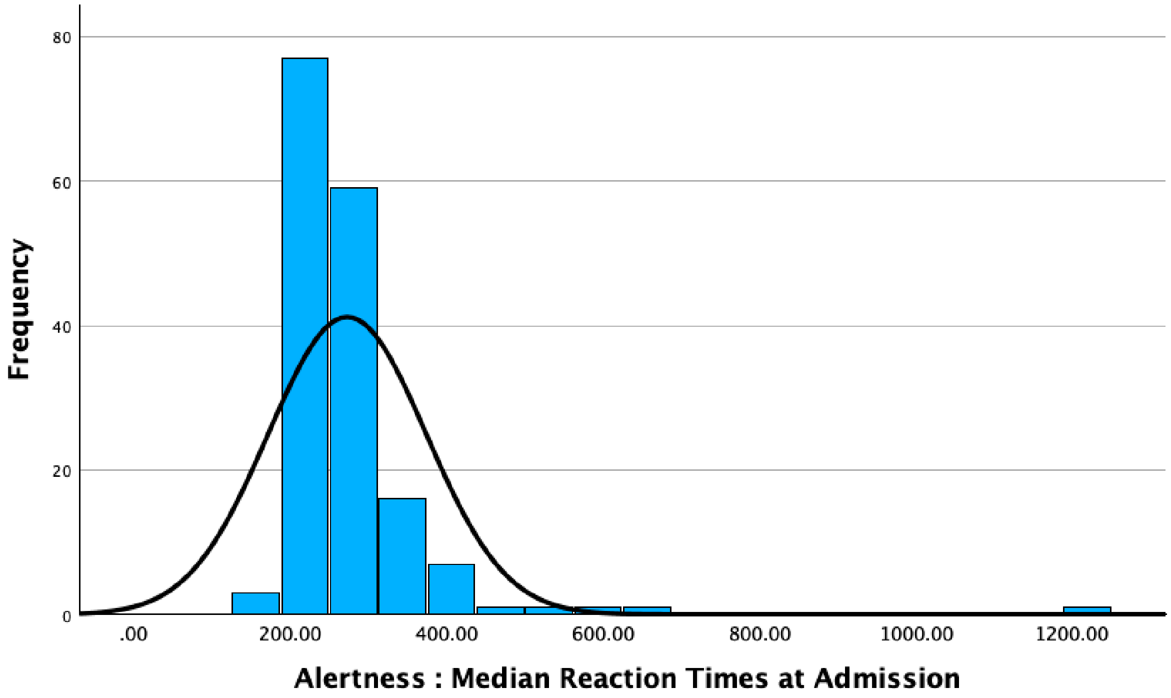

80
60
40
20
0
Frequency
.00
200.00
400.00
600.00
800.00
1000.00
1200.00
Alertness : Median Reaction Times at Admission

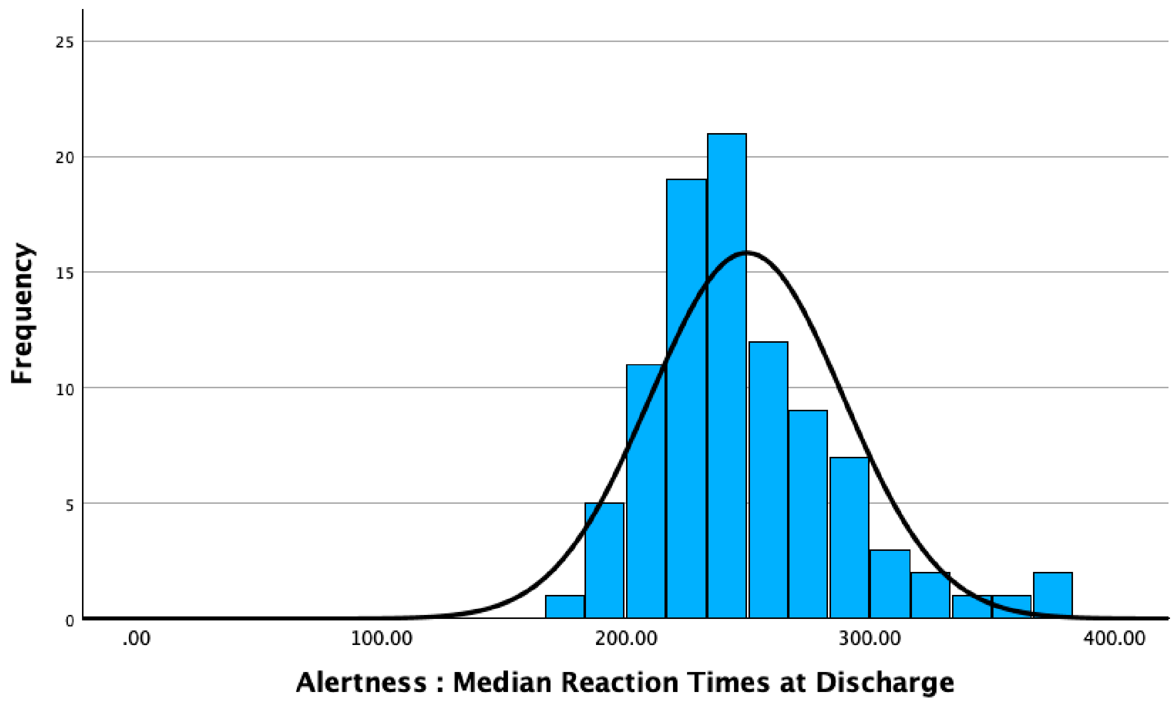
Frequency
25
20
15
10
5
0
.00
100.00
200.00
300.00
400.00
Alertness : Median Reaction Times at Discharge

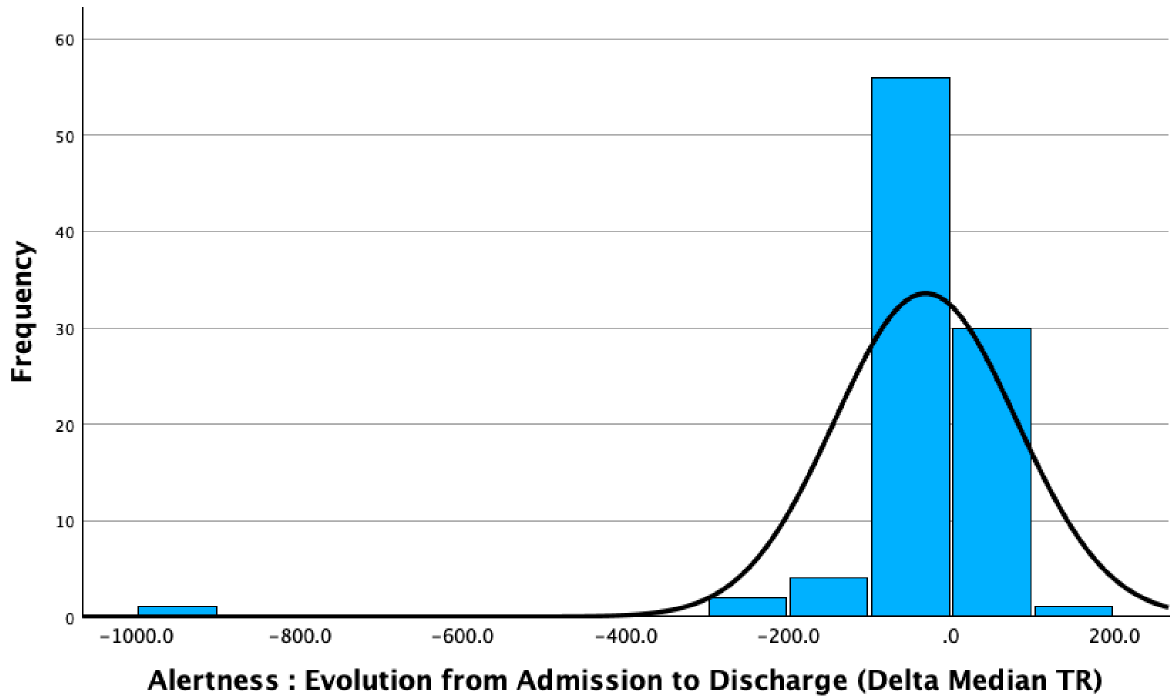
Frequency
60
50
40
30
20
10
0
−1000.0
−800.0
−600.0
−400.0
−200.0
.0
200.0
Alertness : Evolution from Admission to Discharge (Delta Median TR)

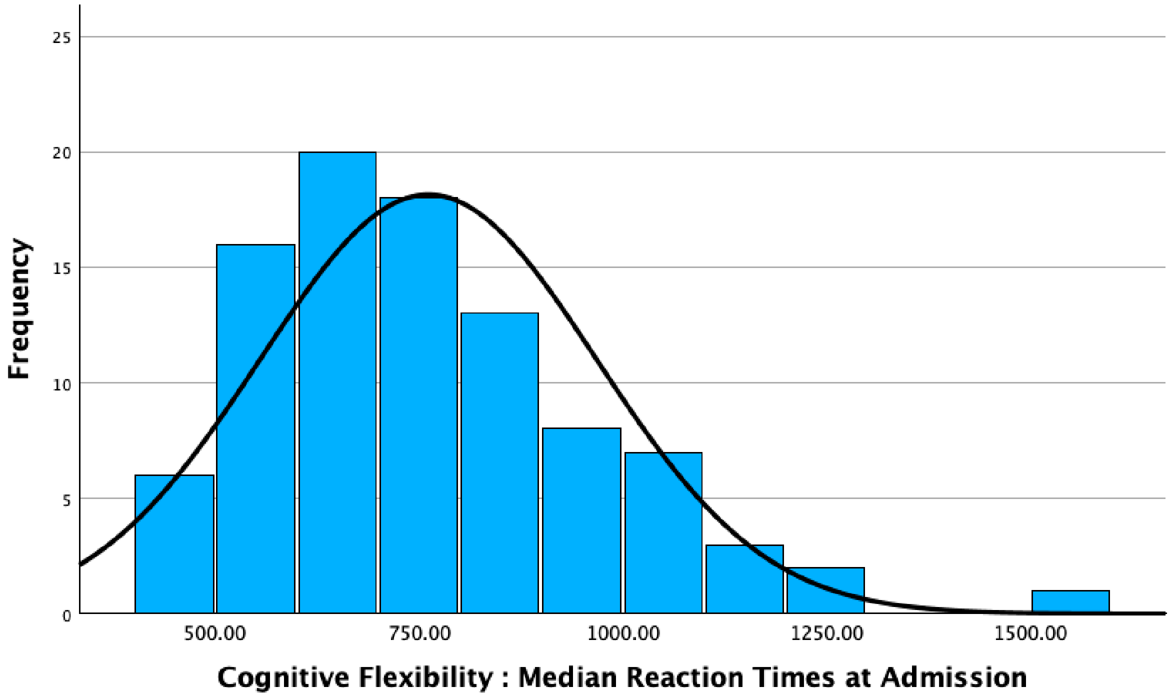

Frequency
25
20
15
10
5
0
500.00
750.00
1000.00
1250.00
1500.00
Cognitive Flexibility : Median Reaction Times at Admission

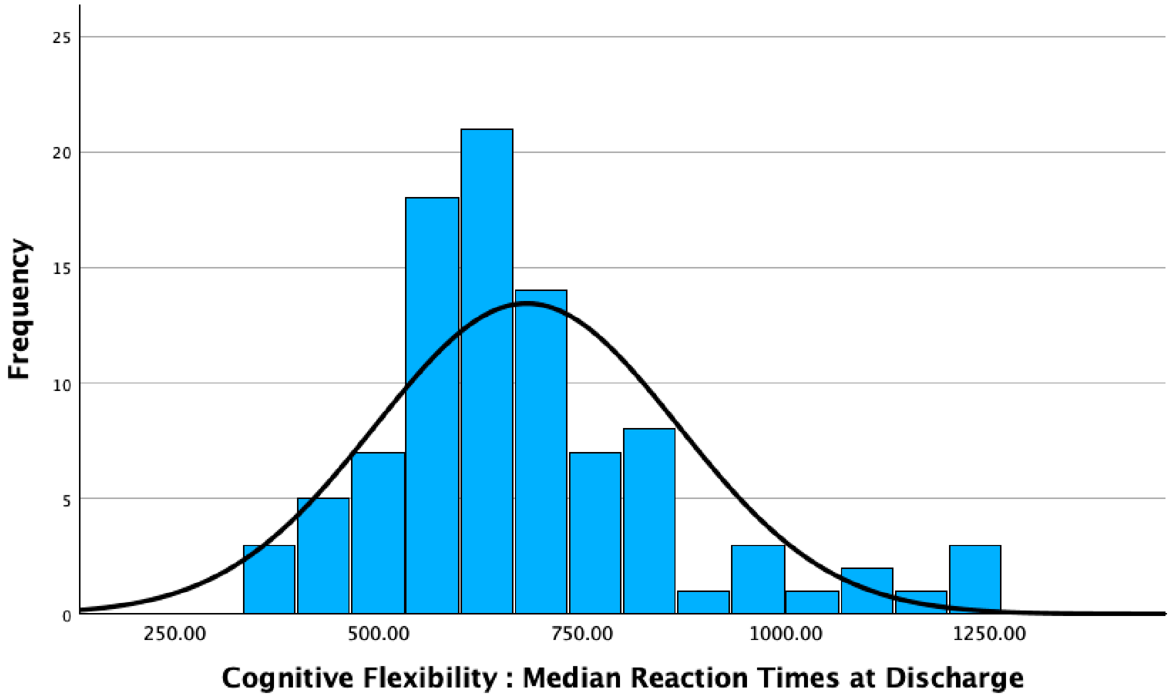

Frequency
25
20
15
10
5
0
250.00
500.00
750.00
1000.00
1250.00
Cognitive Flexibility : Median Reaction Times at Discharge

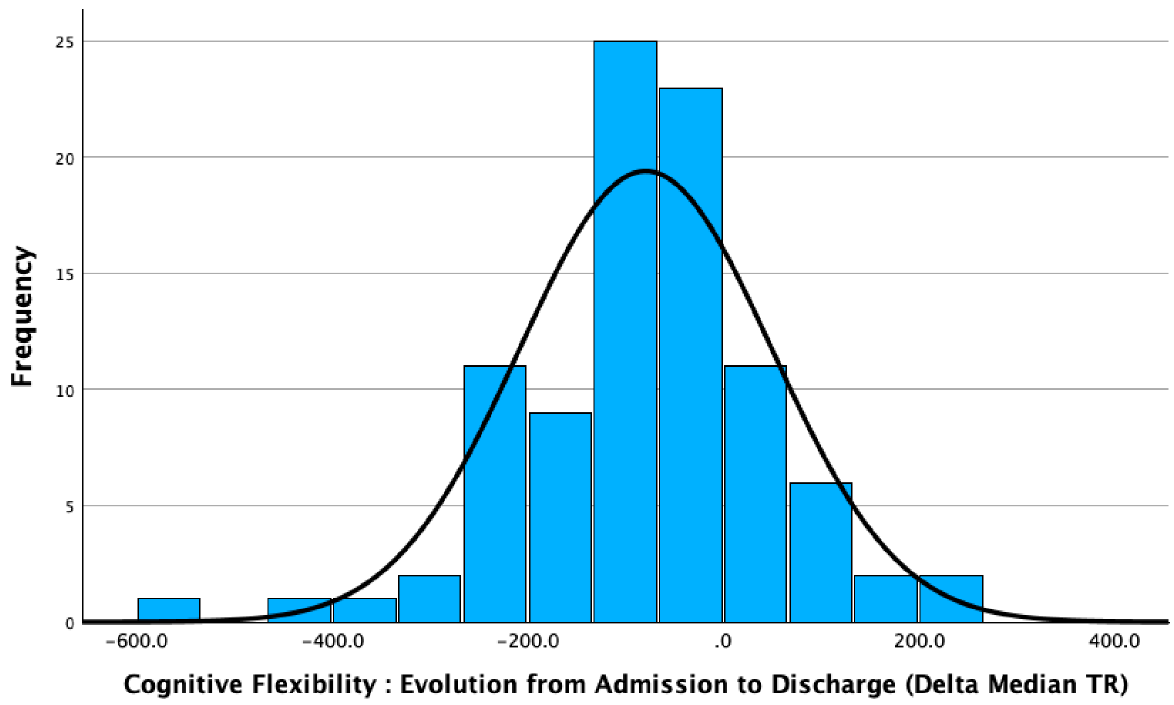
25
20
15
10
5
0
Frequency
-600.0
-400.0
-200.0
.0
200.0
400.0
Cognitive Flexibility : Evolution from Admission to Discharge (Delta Median TR)

Supplementary Table S1:

	Patients at follow-up		Patients lost		Mean comparison
	N	Mean (SD)	N	Mean (SD)	t or z ; p
BMI	94	14.27 (1.29)	73	14.83 (1.69)	2.40 ; **0.015***
FFMI	94	12.53 (0.81)	73	12.62 (0.87)	0.641 ; 0.445
FMI	92	1.78 (0.95)	71	2.29 (1.24)	2.931 ; **0.006***
HAD anx	94	12.83 (4.19)	73	12.25 (4.43)	-0.870 ; 0.561
HAD dep	94	9.26 (4.56)	73	9.23 (4.11)	-0.033 ; 0.286
EDE-Q	94	3.32 (1.37)	73	3.25 (1.25)	-0.573 ; 0.567
Age (years)	94	20.38 (6.03)	73	20.91 (6.76)	-0.566 ; 0.571
Illness duration (years)	94	4.07 (3.96)	73	4.04 (4.06)	-0.545 ; 0.586
Alert. (ms)	94	281.0 (116.0)	73	262.8 (77.5)	-1.752 ; 0.080
Cog. Flex. (ms)	94	761.8 (206.6)	73	740.2 (171.9)	-0.642 ; 0.521

Table S1: Clinical characteristics, self-questionnaires scores and cognitive performances comparisons between complete cases and patients lost to follow up.
BMI: Body Mass Index, FFM: Fat Free Mass, FM: Fat Mass, EDE-Q: Eating Disorders Examination Questionnaire total score, HAD: Hospital Anxiety and Depression scale, Alert.: Alertness performances, Cog. flex. : Cognitive flexibility performances, * Mann-Whitney p<0.05.

Supplementary Table S2:

	Admission				Discharge			
	AN-R Mean (SD)	AN-BP Mean (SD)	Mann-Whitney Z;p	Effect size η^2	AN-R Mean (SD)	AN-BP Mean (SD)	Mann-Whitney Z;p	Effect size η^2
Age (years)	20.60 (5.61)	20.63 (7.06)	-0.693 ; 0.488	0.003	20.53 (5.27)	20.94 (6.63)	-0.121 ; 0.904	<0.001
Illness duration (years)	4.20 (3.82)	3.92 (4.19)	-0.770 ; 0.441	0.004	3.85 (3.12)	4.26 (4.61)	-0.178 ; 0.859	<0.001
BMI	14.94 (1.64)	14.08 (1.21)	-3.501 ; **<0.001***	0.074	17.45 (1.99)	16.88 (2.05)	-1.296 ; 0.195	0.010
FFMI	12.72 (0.91)	12.42 (0.74)	-2.174 ; **0.030***	0.028	13.69 (0.95)	13.44 (1.08)	-1.114 ; 0.265	0.007
FMI	2.28 (1.16)	1.71 (1.00)	-3.230 ; **0.001***	0.063	3.74 (1.28)	3.31 (1.43)	-1.417 ; 0.156	0.012
HAD anx	12.75 (4.23)	12.39 (4.38)	-0.616 ; 0.538	0.002	10.19(3.91)	8.76 (4.33)	-1.657 ; 0.097	0.017
HAD dep	9.55 (4.41)	8.93 (4.31)	-0.654 ; 0.513	0.004	5.12 (3.93)	4.93 (3.81)	-0.248 ; 0.804	<0.001
EDE-Q	3.74 (1.15)	2.82 (1.32)	-4.512 ; **<0.001***	0.123	2.43 (1.36)	1.70 (1.22)	-2.448 ; **0.014***	0.036
Alert. (ms)	265.92(75.81)	280.46(121.98)	-0.829; 0.407	0.004	246.61(36.12)	252.44(42.37)	0.671; 0.502	0.003
Cog. Flex. (ms)	756.6(200.69)	747.90(183.55)	-0.101; 0.920	<0.001	665.80(208.94)	697.4(163.76)	1.273; 0.203	0.010

Table S2: Descriptive and comparative statistics by AN subtype;
AN-R: Anorexia Nervosa Restrictive type, AN-BP: Anorexia Nervosa Binge/Purge type, BMI: Body Mass Index, FFM: Fat Free Mass, FM: Fat Mass, EDE-Q: Eating Disorders Examination Questionnaire total score, HAD: Hospital Anxiety and Depression scale, Alert.: Alertness performances, Cog. flex. : Cognitive flexibility performances. Δ: Delta scores (follow-up minus baseline), * Mann-Whitney p<0.05.

Supplementary Table S3:

	**Age** (years) *Rho;p*	**Illness duration** (years) *Rho;p*	**Number of hospit** *Rho;p*	**BMI** *Rho;p*	**FFM** *Rho;p*	**FM** *Rho;p*	**EDE-Q** *Rho;p*	**HAD anx** *Rho;p*	**HAD dep** *Rho;p*	**Alert.** (ms) *Rho;p*	**Cog. Flex.** (ms) *Rho;p*
Age (years)	-	0.532; **<0.001***	0.105; 0.196	-0.027; 0.731	-0.144; 0.064	0.081; 0.303	0.217; **0.005***	0.197; **0.011***	0.232; **0.003**	0.079; 0.313	0.274; **<0.001***
Illness duration (years)		-	0.369; **<0.001***	-0.109; 0.162	-0.318; **<0.001***	0.068; 0.386	0.213; **0.006***	0.300; **<0.001***	0.247; **0.001***	0.013; 0.865	0.225; **0.003***
Number of hospit			-	-0.239; **0.003***	-0.328; **<0.001***	0.094; 0.251	0.007; 0.929	0.227; **0.005***	0.060; 0.466	-0.018; 0.830	0.121; 0.138
BMI				-	0.658; **<0.001***	0.781; **<0.001***	0.193; **0.012***	-0.080; 0.303	-0.092; 0.235	-0.150; 0.54	-0.063; 0.415
FFM					-	0.116; 0.141	0.211; **0.006** *	-0.066; 0.393	-0.041; 0.602	-0.167; **0.031***	-0.005; 0.953
FM						-	0.090; 0.251	-0.054; 0.495	-0.111; 0.158	-0.033; 0.680	-0.081; 0.303
EDE-Q							-	0.330; **<0.001***	0.358; **<0.001***	-0.061; 0.437	-0.025; 0.751
HAD anx								-	0.459; **<0.001***	0.111; 0.154	0.156; **0.044***
HAD dep									-	0.149; 0.055	0.092; 0.237
Alert. (ms)										-	0.421; **<0.001**

Table S3: Speaman's correlations at baseline
BMI: Body Mass Index, FFM: Fat Free Mass, FM: Fat Mass, EDE-Q: Eating Disorders Examination Questionnaire total score, HAD: Hospital Anxiety and Depression scale, Alert.: Alertness performances, Cog. flex. : Cognitive flexibility performances, * Spearman p<0.05.

Supplementary Table S4:

	**Age** (years) *Rho;p*	**Illness duration** (years) *Rho;p*	**Number of hospit** *Rho;p*	**BMI** *Rho;p*	**FFM** *Rho;p*	**FM** *Rho;p*	**EDE-Q** *Rho;p*	**HAD anx** *Rho;p*	**HAD dep** *Rho;p*	**Alert.** (ms) *Rho;p*	**Cog. Flex.** (ms) *Rho;p*
Age (years)	-	0.586; **<0.001***	0.200; 0.060	- 0.229; **0.026***	-0.331; **0.001***	-0.072; 0.488	0.134; 0.214	0.295; **0.005***	0.291; **0.006***	0.247; **0.017***	0.398; **<0.001***
Illness duration (years)		-	0.319; **0.002***	- 0.223; **0.031***	-0.384; **<0.001***	-0.099; 0.340	0.134; 0.212	0.187; 0.082	0.319; **0.002***	0.102; 0.328	0.201; **0.042***
Number of hospit			-	- 0.057; 0.597	-0.279; **0.008***	0.013; 0.902	0.037; 0.742	0.204; 0.064	0.097; 0383	0.021; 0.846	0.148; 0.168
BMI				-	0.742; **<0.001***	0.881; **<0.001***	- 0.172; 0.109	-0.238; **0.026***	-0.295; **0.005***	-0.368; **<0.001***	-0.241; **0.019***
FFM					-	0.479; **<0.001***	- 0.023; 0.828	-0.219; **0.040***	-0.217; 0.042	-0.407; **<0.001***	-0.298; **0.004***
FM						-	- 0.225; **0.035***	-0.159; 0.138	-0.219; **0.040***	-0.237; **0.021***	-0.141; 0.174
EDE-Q							-	0.612; **<0.001***	0.600; **<0.001***	0.031; 0.774	0.080; 0.459
HAD anx								-	0.667; **<0.001***	0.261; **0.014***	0.189; 0.078
HAD dep									-	0.198; 0.064	0.135; 0.212
Alert. (ms)										-	0.596; **<0.001***

Table S4: Spearman's correlations at follow-up.
BMI: Body Mass Index, FFM: Fat Free Mass, FM: Fat Mass, EDE-Q: Eating Disorders Examination Questionnaire total score, HAD: Hospital Anxiety and Depression scale, Alert.: Alertness performances, Cog. flex. : Cognitive flexibility performances, * Spearman p<0.05.

Supplementary Table S5:

	Admission				Discharge			
**ALL TREATMENTS**	No treatment Mean (SD)	Treatment Mean (SD)	Mann-Whitney Z;p	Effect size η^2	No treatment Mean (SD)	Treatment Mean (SD)	Mann-Whitney Z;p	Effect size η^2
Alert. (ms)	261.74 (54.12)	280.84 (123.13)	0.814; 0.415	0.004	246.81 (45.07)	250.88 (37.29)	1.308; 0.191	0.010
Cog. Flex. (ms)	729.62 (169.49)	767.93 (205.32)	1.116; 0.265	0.008	665.07 (195.30)	689.67 (183.04)	0.727; 0.467	0.003
**ANXIOLYTICS**	No treatment Mean (SD)	Treatment Mean (SD)	Mann-Whitney p	Effect size η^2	No treatment Mean (SD)	Treatment Mean (SD)	Mann-Whitney Z;p	Effect size η^2
Alert. (ms)	262.87 (53.60)	288.27 (144.94)	0.704; 0.482	0.003	249.23 (38.63)	250.46 (41.26)	0.139; 0.889	<0.001
Cog. Flex. (ms)	728.84 (168.44)	787.39 (219.12)	1.626; 0.104	0.016	656.81 (159.84)	722.35 (216.5)	1.289; 0.198	0.010
**ANTIDEPRESSANTS**	No treatment Mean (SD)	Treatment Mean (SD)	Mann-Whitney Z;p	Effect size η^2	No treatment Mean (SD)	Treatment Mean (SD)	Mann-Whitney Z;p	Effect size η^2
Alert. (ms)	272.64 (111.48)	273.83 (79.55)	0.563; 0.574	0.002	248.65 (42.75)	251.02 (35.47)	0.829; 0.407	0.004
Cog. Flex. (ms)	728.27 (189.26)	796.37 (190.53)	2.573; **0.01**	0.040	665.63 (173.69)	703.62 (200.15)	0.707; 0.479	0.003
**ANTIPSYCHOTICS**	No treatment Mean (SD)	Treatment Mean (SD)	Mann-Whitney Z;p	Effect size η^2	No treatment Mean (SD)	Treatment Mean (SD)	Mann-Whitney Z;p	Effect size η^2
Alert. (ms)	273.95 (107.27)	267.88 (56.52)	0.377; 0.706	<0.001	241.05 (37.16)	263.05 (39.69)	3.069; **0.002***	0.057
Cog. Flex. (ms)	732.35 (182.92)	862 (209.91)	3.265; **0.001***	0.064	651.82 (182.70)	730.03 (183.19)	2.620; **0.009***	0.041
**HYPNOTICS**	No treatment Mean (SD)	Treatment Mean (SD)	Mann-Whitney Z;p	Effect size η^2	No treatment Mean (SD)	Treatment Mean (SD)	Mann-Whitney Z;p	Effect size η^2
Alert. (ms)	275.51 (106.95)	261.87 (68.68)	-0.780; 0.436	0.003	251.11 (40.06)	229.17 (22.61)	-1.361; 0.173	0.011
Cog. Flex. (ms)	733.26 (169.84)	839.40 (256.96)	1.914; 0.056	0.022	683.0 (185.83)	676.83 (204.90)	-0.557; 0.578	0.002
**BENZODIAZPINES**	No treatment Mean (SD)	Treatment Mean (SD)	Mann-Whitney Z;p		No treatment Mean (SD)	Treatment Mean (SD)	Mann-Whitney Z;p	
Alert. (ms)	271.27 (105.80)	278.23 (87.07)	0.769; 0.442	0.004	249.89 (43.09)	249.13 (24.96)	0.585; 0.559	0.002
Cog. Flex. (ms)	719.67 (162.09)	846.51 (237.54)	3.239; **0.001***	0.063	658.56 (173.59)	761.32 (206.69)	2.090; **0.037***	0.026

Table S5: Association between treatment (with treatment vs no treatment) and neuropsychological performances Alert.: Alertness performances, Cog. flex. : Cognitive flexibility performances, * Mann-Whitney $p<0.05$.

Supplementary Rank-order linear regressions : Models Summary

BMI: Body Mass Index, FFM: Fat Free Mass, FM: Fat Mass

- **Supplementary Model 1: Linear regression with benzodiazepine treatment, ranked ordered BMI, alertness performances, Age and cognitive flexibility performances at follow-up.**

Model summary

Model	R	R-squared	R-squared adjusted	Estimation Standard Error
1	,659[a]	,434	,409	26,301127

a. Predictors : (Constant), Benzodiazepine treatment, Alertness, Age, BMI

Coefficients[a]

Model		Unstandardized coefficients B	Standard Error	Standardized coefficients Beta	t	Sig.
1	(Constant)	7,424	10,060		,738	,462
	BMI	,024	,061	,034	,390	,697
	Alertness	,543	,088	,538	6,205	<,001
	Age	,170	,061	,242	2,781	,007
	Benzodiazepine treatment	9,346	6,772	,116	1,380	,171

a. Dependent variable : Cognitive flexibility performances

- **Supplementary Model 2: Linear regression with benzodiazepine treatment, ranked ordered FFM, alertness performances, Age and cognitive flexibility performances at follow-up.**

Models summary

Model	R	R-squared	R-squared adjusted	Estimation Standard Error
2	,658[a]	,433	,408	26,314720

a. Predictors : (Constant), Benzodiazpeine treatment, Alertness, Age, FFM

Coefficients[a]

Model		Unstandardized coefficients B	Standard error	Standardized coefficients Beta	t	Sig.
2	(Constant)	8,337	10,710		,778	,438
	FFM	,028	,114	,022	,246	,807
	Alertness	,540	,089	,536	6,059	<,001
	Age	,170	,062	,242	2,744	,007
	Benzodiazepine treatment	9,247	6,785	,115	1,363	,176

a. Dependent variable: Cognitive flexibility performances

- **Supplementary Model 3: Linear regression with benzodiazepine treatment, ranked ordered FM, alertness performances, Age and cognitive flexibility performances at follow-up.**

Model summary

Model	R	R-squared	R-squared adjusted	Estimation Standard Error
3	,658[a]	,433	,408	26,317938

a. Predictors : (Constant), Benzodiazepine treatment, Alertness, FM, Age

Coefficients[a]

		Unstandardized coefficients		Standardized coefficients		
Model		B	Standard error	Beta	t	Sig.
3	(Constant)	9,228	8,918		1,035	,304
	FM	,020	,104	,016	,196	,845
	Alertness	,536	,085	,531	6,276	<,001
	Age	,166	,061	,237	2,748	,007
	Benzodiazepine treatment	9,175	6,769	,114	1,356	,179

a. Dependent variable: Cognitive flexibility performances

- **Supplementary Model 4: Linear regression with antipsychotic treatment, ranked ordered BMI, alertness performances, Age and cognitive flexibility performances at follow up.**

Model summary

Model	R	R-squared	R-squared adjusted	Estimation Standard Error
4	,653[a]	,426	,400	26,488510

a. Predicteurs : (Constant), Antipsychotic treatment, Age, BMI, Alertness

Coefficients[a]

		Unstandardized coefficients		Standardized coefficients		
Model		B	Standard error	Beta	t	Sig.
4	(Constant)	8,059	10,133		,795	,429
	BMI	,018	,061	,026	,300	,765
	Alertness	,518	,091	,513	5,697	<,001
	Age	,189	,059	,270	3,196	,002
	Antipsychotic treatment	4,703	5,959	,068	,789	,432

a. Dependent variable: Cognitive flexibility performances

- **Supplementary Model 5: Linear regression with Antipsychotic treatment, ranked ordered FFM, alertness performances, Age and cognitive flexibility performances at follow-up.**

Model summary

Model	R	R-squared	R-squared adjusted	Estimation Standard Error
5	,652[a]	,425	,399	26,501370

a. Predictors : (Constant), Antipsychotic treatment, FFM, Age, Alertness

Coefficients[a]

		Unstandardized coefficients		Standardized coefficients		
Model		B	Standard error	Beta	t	Sig.
5	(Constant)	9,877	10,710		,922	,359
	FFM	,007	,114	,005	,060	,952
	Alertness	,513	,093	,508	5,498	<,001
	Age	,187	,060	,267	3,102	,003
	Antipsychotic treatment	4,527	5,934	,065	,763	,448

a. Dependent variable: Cognitive flexibility performances

- **Supplementary Model 6: Linear regression with Antipsychotic treatment, ranked ordered FM, alertness performances, Age and cognitive flexibility performances at follow-up.**

Model summary

Model	R	R-squared	R-squared ajusted	Estimation Standard Error
6	,652[a]	,425	,400	26,500252

a. Predictors : (Constant), Neuroleptic treatment, FM, Age, Alertnes

Coefficients[a]

		Unstandardized coefficients		Standardized coefficients		
Model		B	Standard error	Beta	t	Sig.
6	(Constant)	9,736	8,979		1,084	,281
	FM	,011	,104	,009	,105	,916
	Alertness	,513	,089	,508	5,743	<,001
	Age	,186	,058	,266	3,189	,002
	Antipsychotic treatment	4,578	5,951	,066	,769	,444

a. Dependent variable: Cognitive flexibility performances

Supplementary Tables S6 a & b:

Test Statistics[a]

	BMI	FFM	FM	EDE-Q	Alert.	Cog. Flex
Mann-Whitney U	1962,000	1564,000	1529,000	1574,500	1959,000	1466,500
Wilcoxon W	11415,000	2029,000	10440,000	11027,500	11412,000	10919,500
Z	-,388	-2,047	-1,996	-2,003	-,400	-2,453
Asymp. Sig. (2-tailed)	,698	**,041***	**,046***	**,045***	,689	**,014***

a. Grouping Variable: Age (cerebral maturity threshold): less than 25 years old versus 25 years old and above

Table S6a: Comparison by Age group (adolescents vs adults) of nutritional markers, EDE-Q and neuropsychological performances at baseline.
BMI: Body Mass Index, FFM: Fat Free Mass, FM: Fat Mass, EDE-Q: Eating Disorders Examination Questionnaire total score, Alert.: Alertness performances, Cog. flex. : Cognitive flexibility performances, * Mann-Whitney p<0.05.

Test Statistics[a]

	BMI	FFM	FM	EDE-Q	Alert.	Cog. Flex.
Mann-Whitney U	678,000	560,000	590,000	581,500	671,000	502,000
Wilcoxon W	849,000	731,000	3516,000	3066,500	842,000	3428,000
Z	-,058	-1,192	-,903	-,502	-,125	-1,749
Asymp. Sig. (2-tailed)	,954	,233	,366	,616	,901	,080

a. Grouping Variable: Age (cerebral maturity threshold): less than 25 years old versus 25 years old and aboveans

Table S6b: Comparison by Age (adolescents vs adults) of nutritional markers, EDE-Q and neuropsychological performances at follow up.
BMI: Body Mass Index, FFM: Fat Free Mass, FM: Fat Mass, EDE-Q: Eating Disorders Examination Questionnaire total score, Alert.: Alertness performances, Cog. flex. : Cognitive flexibility performances.

EVHAN Group

Jeanne Duclos[1,2,3], Christophe Lalanne[2], Hélène Roux[1,2], Marie Raphaële Thiébaud[1,2], Sarah Vibert[1,2], Tamara Hubert[2], Annaig Courty[1,4], Damien Ringuenet[4], Jean-Pierre Benoit[1,5], Corinne Blanchet[1,5], Marie Rose Moro[1,5], Laura Bignami[6], Clémentine Nordon[6], Frédéric Rouillon[6,7], Solange Cook[8], Catherine Doyen[6,8], Marie-Christine Mouren Siméoni[6], Priscille Gerardin[9], Sylvie Lebecq[9], Marc-Antoine Podlipski[9], Claire Gayet[9], Malaika Lasfar[9], Marc Delorme[10], Xavier Pommereau[10], Stéphanie Bioulac[10,11], Manuel Bouvard[10,12], Jennifer Carrere[10], Karine Doncieux[13], Sophie Faucher[13], Catherine Fayollet[13], Amélie Prexl[13], Stéphane Billard[14,15], François Lang[14,15], Virginie Mourier-Soleillant[14], Régine Greiner[14], Aurélia Gay[14,15], Guy Carrot[14,15], Sylvain Lambert[16], Morgane Rousselet[16,17], Ludovic Placé[16,17], Jean-luc Venisse[16,17], Marie Bronnec[16,17], Bruno Falissard[1], Christophe Genolini[18], Christine Hassler[1], Jean-Marc Tréluyer[19], Olivier Chacornac[1], Maryline Delattre[19], Nellie Moulopo[19], Christelle Turuban[19] and Christelle Auger[19].

1: CESP, INSERM, University Paris-Sud, UVSQ, University Paris-Saclay, Paris, France

2: Institut Mutualiste Montsouris, Paris, France

3: University of Reims, EA 6291, Reims, France

4: Hospital Paul Brousse, AP-HP, Villejuif, France

5: Maison de Solenn, Hospital Cochin, AP-HP, Paris, France

6: CMME, Saint Anne Hospital, Paris, France

7: INSERM Center 894, Paris, France

8: Hospital Robert Debré, AP-HP, Paris, France

9: University Hospital of Rouen, France

10: University Hospital of Bordeaux, Bordeaux, France

11: USR CNRS 3413 SANPSY, Bordeaux, France

12: Bordeaux University, Bordeaux, France

13: Institut Marcel Rivière, La Verrière, Le Mesnil Saint-Denis, France

14: University Hospital of Nord, Saint-Etienne, France

15: University of Saint-Etienne, EA 4556 laboratory Epsylon, France

16: University Hospital of Nantes, Nantes, France

17: University of Nantes, EA 4275, France

18: UMR 1027, Toulouse, France

19: URC-CIC Cochin Necker, AP-HP, Paris, France.
